# Plasmonic Nanostructures for Exosome Biosensing: Enabling High-Sensitivity Diagnostics

**DOI:** 10.3390/nano15151153

**Published:** 2025-07-25

**Authors:** Seungah Lee, Nayra A. M. Moussa, Seong Ho Kang

**Affiliations:** 1 Department of Applied Chemistry and Institute of Natural Sciences, Kyung Hee University, Yongin-si 17104, Gyeonggi-do, Republic of Korea; moon1131@gmail.com; 2 Department of Chemistry, Graduate School, Kyung Hee University, Yongin-si 17104, Gyeonggi-do, Republic of Korea; nayramoussa8@gmail.com

**Keywords:** exosome, nanoplasmonic biosensors, propagating surface plasmon resonance, localized surface plasmon resonance, surface-enhanced Raman scattering, high-sensitivity diagnostics

## Abstract

Exosomes are nanoscale extracellular vesicles (EVs) that carry biomolecular signatures reflective of their parent cells, making them powerful tools for non-invasive diagnostics and therapeutic monitoring. Despite their potential, clinical application is hindered by challenges such as low abundance, heterogeneity, and the complexity of biological samples. To address these limitations, plasmonic biosensing technologies—particularly propagating surface plasmon resonance (PSPR), localized surface plasmon resonance (LSPR), and surface-enhanced Raman scattering (SERS)—have been developed to enable label-free, highly sensitive, and multiplexed detection at the single-vesicle level. This review outlines recent advancements in nanoplasmonic platforms for exosome detection and profiling, emphasizing innovations in nanostructure engineering, microfluidic integration, and signal enhancement. Representative applications in oncology, neurology, and immunology are discussed, along with the increasingly critical role of artificial intelligence (AI) in spectral interpretation and diagnostic classification. Key technical and translational challenges—such as assay standardization, substrate reproducibility, and clinical validation—are also addressed. Overall, this review highlights the synergy between exosome biology and plasmonic nanotechnology, offering a path toward real-time, precision diagnostics via sub-femtomolar detection of exosomal miRNAs through next-generation biosensing strategies.

## 1. Introduction

Exosomes, small EVs ranging from 30 to 200 nm in diameter [1,2], facilitate intercellular communication by transferring biomolecules such as proteins, lipids, and RNA [3]. Released by various cell types, exosomes play critical roles in physiological and pathological processes, including immune responses [4,5], tissue repair [6,7], and modulation of the tumor microenvironment [8]. Due to their ability to encapsulate and transport molecular signatures reflective of their parent cells, exosomes have gained recognition as promising biomarkers for early disease detection, prognosis, and therapeutic monitoring [9,10]. Despite this potential, their clinical application remains limited due to challenges in characterization using conventional analytical methods. Their small size, heterogeneity, and the complexity of biological environments complicate traditional isolation and analysis protocols, often lacking the sensitivity and specificity required for single-vesicle resolution.

While fluorescence-based bead assays are commercially available, these methods are inadequate for single-exosome quantification and necessitate labeling, which may introduce artifacts and affect the native state of the exosomes. Conventional optical and imaging techniques, such as dynamic light scattering (DLS) [11], nanoparticle tracking analysis (NTA) [12], flow cytometry (FCM) [13], transmission electron microscopy (TEM) [14], and atomic force microscopy (AFM) [15,16,17,18,19], have been utilized for exosome characterization, although each presents specific limitations. These methods are discussed in greater detail in Section 3.

To overcome these limitations, researchers are increasingly turning to innovative biosensing approaches. Among these, nanoplasmonic biosensors—particularly PSPR, LSPR, and SERS—have shown remarkable potential due to their high sensitivity, label-free operation, and single-vesicle resolution. The principles of these technologies are discussed in Section 4, followed by representative applications in Section 5. To our knowledge, few reviews have systematically explored the integration of plasmonic biosensing strategies for exosome profiling with emerging AI-assisted spectral analysis techniques. This review aims to provide a timely, application-oriented perspective that complements and extends beyond the prior general plasmonic literature.

## 2. Exosomal Biomarkers

### 2.1. Origin of Exosomes

Exosomes are small EVs derived from multivesicular bodies (MVBs) and are characterized by their distinctive disc- or cup-shaped morphology [20]. Once formed by various cell types, exosomes are released into and circulate through a wide range of bodily fluids, including cerebrospinal fluid (CSF), blood, urine, and saliva. Their biogenesis originates from the endosomal pathway [21], a fundamental mechanism for intracellular trafficking and intercellular communication.

**Figure 1:**
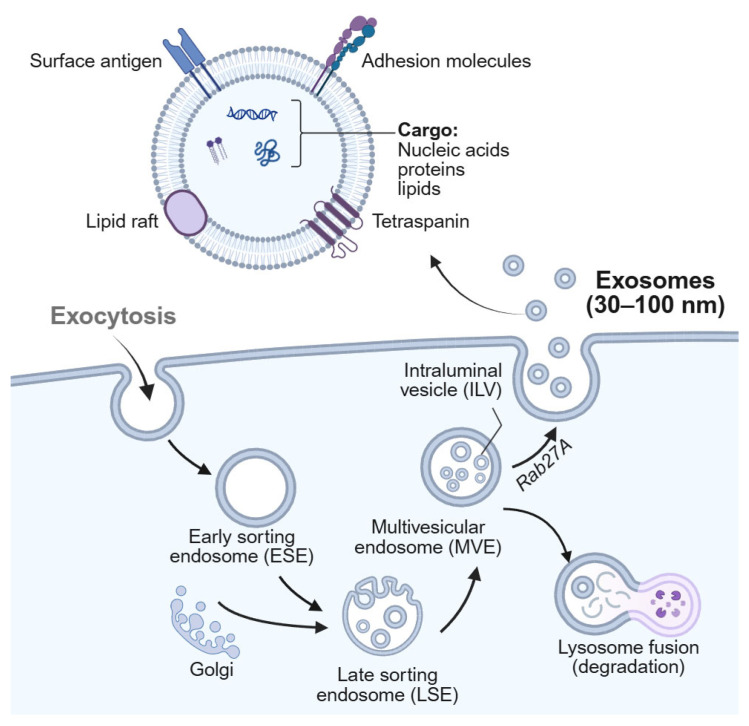
Schematic illustration of the exosome biogenesis and secretion pathway. Created with BioRender.com under an authorized license.

This pathway begins with the invagination of the plasma membrane, resulting in the internalization and encapsulation of cytosolic components to form early sorting endosomes (ESEs). As endosomes mature, their biomolecular composition changes, giving rise to late sorting endosomes (LSEs). During this maturation process, the endosomal membrane further invaginates inward to form intraluminal vesicles (ILVs) within the lumen of the endosome, ultimately resulting in the formation of multivesicular bodies (MVBs). MVBs can follow two main fates: they may fuse with lysosomes for degradation, or alternatively, fuse with the plasma membrane, leading to the release of ILVs into the extracellular space. These secreted ILVs are what we commonly refer to as exosomes [22] (Figure 1).

### 2.2. Classification of Exosomes

Exosomes can be broadly classified based on their cellular origin and the presence or absence of artificial modifications. From this perspective, they are typically divided into two major categories: natural exosomes, which are secreted by unmodified cells, and engineered exosomes, which are intentionally altered to enhance or modify their functional properties [21]. Notably, while mammalian exosomes are EVs formed through a conserved endosomal biogenesis pathway and characterized by specific biomarkers such as tetraspanins, exosome-like nanoparticles (ELNs) derived from plants and food sources do not share these biogenesis mechanisms or marker profiles. This distinction means that, despite similarities in size and morphology, ELNs represent a separate class of vesicles and should not be classified as true exosomes [23].

#### 2.2.1. Mammalian Exosomes

Exosomes can be secreted by virtually all mammalian cells under both physiological and pathological conditions. Common sources include human umbilical vein endothelial cells (HUVECs), mesenchymal stem cells (MSCs), macrophages, and natural killer (NK) cells [24,25,26]. These vesicles inherit a unique molecular cargo reflective of their parent cells, contributing to their heterogeneity in composition, size, and function [27]. Cell type significantly affects the yield, biomolecular content, immunological role, and drug-loading capacity of the exosomes. For instance, stem cell-derived exosomes often demonstrate high production efficiency and efficient drug encapsulation, while macrophage-derived exosomes exhibit potent immunomodulatory and anti-tumor activity [28]. Animal-derived exosomes are also abundant in various biofluids, such as saliva, plasma, urine, ascitic fluid, milk, and bile. Among these, milk-derived exosomes have garnered attention as drug delivery carriers owing to their stability, biocompatibility, and low immunogenicity. Notably, paclitaxel-loaded milk exosomes have shown promise in preclinical models of cancer therapy [29].

#### 2.2.2. Engineered and Functionalized Exosomes

While natural and exosome-like vesicles derived from animals or plants exhibit a variety of promising biofunctional properties, they are inherently limited by factors such as low production yield, heterogeneous composition, and nonspecific biodistribution. To address these challenges, engineered exosomes have been developed through advanced techniques such as genetic modification, surface functionalization, and therapeutic cargo loading [30,31,32]. These modifications aim to enhance targeting specificity, circulatory stability, and biological efficacy. Engineered exosomes can be tailored to deliver a range of therapeutic payloads—including small molecules, nucleic acids, proteins, and imaging agents—to specific tissues or cell types with high precision. Recent studies have demonstrated their potential in targeted cancer therapy, gene editing applications (e.g., CRISPR/Cas9 delivery), and vaccine development [33,34,35,36]. Importantly, synthetic strategies offer a practical means to overcome the variability and delivery limitations associated with naturally derived vesicles, thereby expanding the therapeutic utility of exosome-based systems.

#### 2.2.3. Emerging Functional Taxonomies

Traditional classification frameworks for exosomes have primarily relied on the cell or tissue of origin, which, although informative, are insufficient to capture the functional diversity and therapeutic potential of exosomes. This limitation becomes particularly evident in the context of translational research, where understanding the dynamic biological behavior of exosomes is essential for their effective clinical application [21].

In response, a growing body of research advocates for functionally informed and application-oriented classification systems that better reflect the biological roles of exosomes and guide their rational design in biomedical settings. One emerging criterion is organotropism, which describes the natural tendency of exosomes to accumulate in specific target organs—such as the brain, liver, or lungs—making it a valuable parameter for organ-specific drug delivery strategies. Another is systemic biodistribution, which refers to the in vivo circulation, clearance, and tissue retention of exosomes following administration. Additionally, the immunological profile of exosomes—including both their immunogenic potential and capacity for immune modulation—is increasingly recognized as a critical determinant for therapeutic efficacy and safety [37].

Complementing these functional parameters, recent advances in single-vesicle profiling, omics-based phenotyping, and AI-driven clustering algorithms are facilitating the development of multi-dimensional classification frameworks. These integrative approaches combine intrinsic properties of exosomes—such as size, membrane composition, and molecular cargo—with extrinsic biological behaviors, including biodistribution, cellular uptake, and therapeutic performance.

In summary, moving beyond source-based classification toward a functionally grounded, application-driven taxonomy represents a crucial shift in unlocking the full diagnostic and therapeutic potential of exosomes in precision medicine.

## 3. Exosome Detection Techniques

### 3.1. Conventional Analytical Methods

Exosome detection and characterization have traditionally relied on a variety of biochemical and physical techniques. These include Western blotting [38], enzyme-linked immunosorbent assays (ELISAs) [39], mass spectrometry (MS) [40], NTA [41], DLS [11], FCM [13], TEM [14], and AFM [15]. These techniques have been indispensable in establishing the foundational knowledge of exosomal size, composition, and morphology.

However, despite their contributions, these methods present significant limitations for clinical translation. DLS and NTA are ensemble techniques that struggle with polydisperse samples and require careful calibration. For example, while DLS can theoretically detect vesicles below 70 nm, its accuracy is compromised in polydisperse samples due to ensemble averaging effects. FCM, despite its high throughput, remains limited in detecting exosomes below 100 nm without advanced instrumentation and fluorescent labeling strategies. TEM and AFM provide high-resolution imaging but require elaborate sample preparation and lack scalability for clinical diagnostics. Likewise, Western blot and ELISAs offer protein-specific detection but are labor-intensive and do not support single-exosome resolution. These shortcomings underscore the need for more sensitive, label-free, and high-throughput alternatives for clinical-grade exosome analysis.

### 3.2. Emerging Biosensing Approaches

To overcome the limitations of conventional methods, recent efforts have focused on developing biosensing platforms capable of highly sensitive, rapid, and quantitative exosome detection. These approaches are designed to function effectively in complex biological fluids and ideally provide label-free, real-time analysis at the single-vesicle level.

Among the emerging strategies, optical biosensors, electrochemical sensors, and interferometric imaging platforms have shown particular promise. One notable example is single-particle interferometric reflectance imaging sensing (SP-IRIS), which utilizes light interference patterns to detect nanoscale vesicles without the need for labels [42]. SP-IRIS achieves high sensitivity for particles as small as ~50 nm and allows multiplexed detection using antibody-functionalized surfaces.

Another promising direction is the integration of biosensors with microfluidic chips, enabling automated sample processing, enrichment, and detection within compact and cost-effective formats. These lab-on-a-chip devices facilitate rapid turnaround times and minimal sample consumption—essential features for clinical point-of-care (POC) applications. When integrated with microfluidic or optical platforms, AI improves the accuracy and robustness of exosome classification, enabling insights into their origin, cargo, and disease relevance.

Overall, these biosensing strategies offer a transformative step toward real-time, clinically relevant exosome diagnostics and serve as a foundation for integrating nanoplasmonic technologies, which are discussed in the following section.

## 4. Nanoplasmonic Biosensors for Exosome Detection

### 4.1. Principles of Plasmonics in Biosensing

Nanoplasmonics leverages the unique optical phenomena that arise from the interaction between light and metallic nanostructures, serving as a next-generation diagnostic tool capable of detecting biomolecules with exceptional sensitivity. This is particularly advantageous for detecting nanoscale EVs such as exosomes, which are typically low in abundance and small in size. Nanoplasmonic platforms offer a powerful solution to overcome the limitations of conventional analytical techniques [43,44].

At the core of this technology lies SPR, where incident light resonates with collective oscillations of free electrons at a metal surface, creating an enhanced electromagnetic field. SPR can be categorized into two major types based on its propagation characteristics: PSPR and LSPR. These modalities differ significantly in terms of their detection depth, sensitivity to bulk refractive index changes, and suitability for miniaturized biosensing platforms [45,46]. Specifically, PSPR offers a deeper penetration depth (~200–300 nm), making it ideal for detecting bulk refractive index changes in complex media, while LSPR confines its enhanced electromagnetic field within ~10–30 nm of the nanostructure surface, enabling higher sensitivity to molecular binding events occurring in close proximity to the sensor (Table 1). Notably, recent advances have demonstrated the synergistic enhancement of SERS signals through the deliberate coupling of PSPR and LSPR modes. Ryu et al. engineered a crater-structured substrate on a GaAs wafer coated with silver nanowires (AgNWs) to achieve this effect [47]. In this system, the crater structure supports PSPR, while the AgNWs generate LSPR. The interaction between these two plasmonic modes substantially amplifies the local electromagnetic field, resulting in SERS intensity enhancements of up to 17.4-fold compared to conventional flat surfaces without AgNWs. This enhancement was validated through both experimental Raman measurements and finite-difference time-domain (FDTD) simulations, confirming the effectiveness of the PSPR–LSPR coupling strategy. The underlying mechanism is attributed to strong electromagnetic fields formed at the interface between the metallic crater and densely packed AgNWs, where “hot spots” arise from the effective coupling of propagating and localized plasmons. This approach not only increases local field intensity but also improves the uniformity and reproducibility of SERS signals. The findings suggest that further optimization of crater geometry and nanowire density could maximize plasmonic coupling, offering a promising route for the development of highly sensitive and reliable SERS substrates for chemical and biological sensing applications, including exosome detection.

Building on advances in engineered plasmonic substrates, recent theoretical work has explored the potential of 2D materials for tunable plasmonic coupling. Pan et al. investigated a borophene-based metamaterial, demonstrating strong coherent coupling between borophene surface plasmon modes and borophene localized surface plasmon modes within a grating structure [48]. By adjusting the carrier density of the borophene gratings, the researchers achieved dynamic control over the hybridization of these modes, resulting in multiple hybrid plasmonic states and a pronounced Rabi splitting with a frequency of 21.6 THz. FDTD simulations and a coupled oscillator model confirmed that this coupling produces nearly perfect, tunable absorption peaks, highlighting the remarkable light–matter interaction achievable in borophene systems. This study further revealed that the strength of coherent coupling—and thus the optical response—can be modulated by varying the distance between the periodic borophene nanoribbons and the continuous borophene sheet, as well as by changing the incident angle and relaxation time. Importantly, the inclusion of a gold substrate significantly enhanced absorption efficiency by acting as a reflective mirror, thereby strengthening surface plasmon resonances. These findings not only deepen the understanding of plasmon hybridization in emerging 2D materials but also suggest practical strategies for designing actively tunable, high-performance photonic and optoelectronic devices in the near-infrared region, with promising implications for future biosensing and environmental monitoring applications.

### 4.2. PSPR-Based Technology

PSPR operates on planar metallic thin films (typically gold or silver), where surface plasmons propagate laterally along the interface. Excitation is achieved using P-polarized light coupled via a prism or grating, generating an evanescent field that penetrates approximately 200 nm into the adjacent dielectric medium [45].

When exosomes bind to capture ligands immobilized on the sensor surface, they induce local refractive index changes within the penetration depth of this field. This leads to shifts in the reflected light’s angle, intensity, or wavelength, allowing for real-time, label-free detection of molecular interactions. PSPR platforms—especially SPR imaging (SPRi)—enable multiplexed and quantitative analyses of surface-bound exosomes with high sensitivity. Due to their deeper penetration depth and higher sensitivity to bulk changes, PSPR sensors are particularly well suited for analyzing exosomes in complex biological samples such as serum or cell culture media [49,50].

### 4.3. LSPR-Based Technology

LSPR involves the excitation of localized surface plasmons on metallic nanostructures (e.g., nanospheres, nanorods, nanoshells), producing strong, confined electromagnetic fields in the immediate vicinity of the nanostructure surface [46,51,52]. These fields are extremely sensitive to local changes, such as biomolecular binding events, making LSPR a powerful approach for label-free biosensing.

Unlike PSPR, LSPR does not require prism coupling and can be implemented in compact, cost-effective formats. Recent advances in LSPR sensor platforms include the use of nanohole arrays and ordered nanoparticle assemblies, which allow for real-time and high-sensitivity detection of exosomes [43,53,54]. Notably, the nanoplasmonic exosome (nPLEX) assay uses a transmission SPR mode with periodic nanohole arrays to quantitatively analyze both surface and intravesicular proteins [55]. When integrated with portable optical systems, these platforms offer high sensitivity, multiplexing, and miniaturization—critical features for POC diagnostics [56].

### 4.4. SERS-Based Technology

SERS builds upon the principles of LSPR by amplifying inherently weak Raman signals through the formation of plasmonic “hot spots” near metallic nanostructures. While typical SERS substrates yield enhancement factors ranging from 10^6^ to 10^8^, optimized architectures have demonstrated amplification of up to 10^10^-fold, enabling the acquisition of rich, molecule-specific vibrational spectra and even single-molecule detection under ideal conditions [51,57] (Table 1).

While LSPR-based sensors are ideal for quantitative and kinetic analyses based on refractive index changes, SERS enables detailed molecular fingerprinting of exosomes. SERS platforms support both label-free detection, by directly probing intrinsic exosomal contents (e.g., lipids, nucleic acids) and label-based assays using SERS tags composed of Raman reporters conjugated to targeting ligands. These platforms offer ultrasensitive detection—including single-exosome resolution—and multiplexed analysis of multiple exosomal biomarkers within a single measurement.

Despite these advantages, SERS still faces technical limitations such as variability in hotspot distribution, challenges in substrate reproducibility, and the complexity of spectral data interpretation. These issues remain key barriers to its widespread clinical translation.

### 4.5. Clinical Applications and Technological Integration

Nanoplasmonic biosensors based on PSPR, LSPR, and SERS have evolved into robust platforms capable of precisely analyzing exosomal biomarkers across diverse disease contexts. PSPR is well suited for bulk refractive index-based quantitative interaction studies, while LSPR offers excellent scalability and integration with miniaturized diagnostic devices. SERS provides molecular specificity and high-resolution fingerprinting, facilitating detailed profiling at the single-exosome level.

These technologies have been applied to detect cancer, neurodegenerative, and immune-related diseases. For instance, surface proteins such as HER2, EpCAM, and CD63 on exosomes from patients with breast, lung, and prostate cancers have been sensitively detected using SPR and LSPR platforms [49,53,58]. Additionally, SPRi has enabled the detection of neuron-derived exosomes in plasma, offering a promising approach for early diagnosis of Alzheimer’s disease (AD) and other neurodegenerative conditions [50,59].

Moreover, the integration of LSPR and SERS platforms with microfluidic chips and AI-powered data analysis pipelines is advancing the field toward automated, high-throughput, and quantitative exosome detection. These hybrid systems—especially when coupled with portable optical components—are paving the way for next-generation diagnostic tools that combine sensitivity, specificity, and clinical practicality. The convergence of nanoplasmonics with digital technologies is expanding the utility of exosome biosensors beyond simple detection toward early diagnosis, molecular subtyping, and therapeutic monitoring.

Xie et al. developed an AI-based SERS strategy for label-free spectroscopic analysis of serum exosomes, enabling accurate diagnosis of breast cancer and assessment of surgical outcomes with a focus on clinical trials [60]. The methodology involved training a deep learning algorithm, specifically an artificial neural network, on SERS spectra of exosomes derived from four breast cancer cell lines (MDA-MB-231, MCF-7, BT474, and SKBR-3) to model distinct breast cancer subtypes. This approach was then critically validated in a clinical trial using serum exosomes directly isolated from human patients. A key result from the clinical validation was the demonstration of 100% prediction accuracy for breast cancer patients who had not undergone surgery. Furthermore, in the clinical assessment of surgical outcomes, the integration of this SERS profiling with principal component analysis-based similarity analysis enabled the effective evaluation of breast cancer surgical efficacy across different molecular subtypes. This study showed that successful surgical resection correlated with a reduction in the “relative similarity” of patient serum exosomes to cancer cell exosomes, thereby providing a robust indicator of treatment response.

Song et al. introduced a highly sensitive and precise programmable curved plasmonic nanoarchitecture-based biosensor for the clinical diagnosis of AD by analyzing exosomal microRNAs (exomiRs) in patient serum samples [61]. For their clinical trials, the researchers collected serum samples from three hospital-based cohorts: AD patients, mild cognitive impairment (MCI) patients, and healthy controls (HCs). The method involved isolating exosomes from these serum samples, extracting exomiRs, and then detecting four specific AD-related exomiRs (exomiR-125b, exomiR-135a, exomiR-15a, and exomiR-20a) using their novel plasmonic biosensor, which featured attomolar-level detection limits through enhanced electromagnetic fields in its unique nanostructure. A key result from the clinical application was the successful classification of individuals into AD, MCI, and HC groups, with an average accuracy of 98.22% by profiling and quantifying these exomiRs. Furthermore, integrating the analysis of exomiRs with core AD biomarkers such as Aβ1-42 significantly improved diagnostic performance, achieving an average sensitivity of 95.83%, selectivity of 80.0%, and accuracy of 98.62% for distinguishing HCs from AD patients, and 100.0% sensitivity, 80.0% selectivity, and 100.0% accuracy for MCI patients. This demonstrated the robust capacity of the biosensor for early AD diagnosis and tracking cognitive decline progression in a clinical setting.

Wu et al. developed a dedicated platform for multiparametric exosome analysis, enabling simultaneous biophysical and biomolecular evaluation of the same vesicles directly in clinical biofluids for improved disease prognosis [62]. Termed templated plasmonics for exosomes (TPEX), the technology leverages in situ growth of gold nanoshells on vesicles to achieve multi-selectivity; biophysical selectivity is achieved by templating nanoshell formation to distinguish exosome dimensions, while biomolecular selectivity relies on the nanoshell plasmonics locally quenching fluorescent probes only when they are target-bound on the same vesicle. In their clinical utility evaluation, the researchers applied the TPEX platform to analyze patient ascites samples from colorectal and gastric cancer patients (*n* = 20) using a miniaturized microfluidic, smartphone-based sensor. A key result from these clinical samples was that the TPEX model, which measures exosomal targets, achieved significantly higher accuracy (AUC = 0.970 for both cancers combined) in classifying patient prognosis (good vs. poor survival) compared to conventional ELISA analysis of total target proteins (AUC = 0.758). This demonstrated that the exosomal subpopulation of biomarkers, as specifically measured using TPEX, could more accurately differentiate patient prognosis, highlighting the potential of the platform for superior disease stratification in clinical settings.

Im et al. established a label-free, high-throughput nanoplasmonic exosome (nPLEX) assay for the quantitative analysis and molecular profiling of exosomes, particularly focusing on their potential for cancer diagnostics [63]. The method used the transmission surface plasmon resonance through periodic nanohole arrays, functionalized with antibodies to profile exosome surface proteins. For their clinical trials, the researchers applied the nPLEX assay to analyze ascites samples from ovarian cancer patients (*n* = 20) and non-cancerous cirrhosis patients (*n* = 10) as controls. They also conducted a longitudinal monitoring study on ovarian cancer patients (*n* = 8) undergoing chemotherapy, collecting ascites samples before and after treatment. A key result from the profiling study showed that exosomal EpCAM and CD24 levels were significantly higher in ovarian cancer patient samples, and pairing these protein profiles increased diagnostic accuracy to 97% for distinguishing cancer from non-cancer controls. In the longitudinal study, they observed that exosomal EpCAM and CD24 levels in patients decreased responding to chemotherapy, while increasing in non-responding patients, suggesting the potential of the nPLEX assay for monitoring treatment response, although the cohort size was small for statistical significance. This demonstrates the improved sensitivity and ability of the nPLEX platform to provide rapid, multiplexed analysis of exosomes directly in clinical biofluids.

In summary, PSPR, LSPR, and SERS each offer distinct but complementary advantages. Their integration enables the development of next-generation nanoplasmonic biosensors that are highly sensitive, specific, and clinically translatable. These approaches are converging to build future-ready diagnostic platforms capable of real-time, multiplexed, and personalized exosome analysis, driving forward the promise of precision medicine and theranostics.

## 5. Applications of Nanoplasmonic Biosensors in Exosome Detection

### 5.1. PSPR-Based Platforms

PSPR platforms have gained prominence for their ability to detect exosomes with high sensitivity, label-free operation, and real-time monitoring. The evanescent field generated on planar gold surfaces typically penetrates less than 300 nm into the surrounding medium—comparable to the size of exosomes—making PSPR particularly suitable for studying surface protein interactions on vesicles [43].

A notable example is the label-free SPR biosensor developed by Sina et al., which successfully detected exosomes derived from BT474 breast cancer cells with a detection limit as low as 8280 exosomes/μL [49]. The sensor maintained high analytical performance even in complex biological matrices, such as serum-spiked samples, and effectively addressed nonspecific adsorption through the implementation of alternating-current electrohydrodynamic (ac-EHD) micro-mixing, which enhanced antigen–antibody binding efficiency while simultaneously removing weakly bound biomolecules from the sensing surface.

Expanding the clinical utility of SPR biosensing, Picciolini et al. developed a SPRi-based antibody microarray capable of isolating and characterizing multiple neural-derived exosome subpopulations directly from human plasma [50]. The system achieved simultaneous, label-free quantification of exosomes originating from neurons and oligodendrocytes using only 500 μL of plasma and commercially available reagents, with sensitivity and specificity comparable to conventional ELISAs. Notably, the platform allowed sequential antibody injection to assess varying membrane marker levels across different exosome subsets, enabling fine-resolution phenotyping of brain-derived EVs. This approach highlights the potential of SPRi for non-invasive diagnosis and longitudinal monitoring of neurodegenerative and central nervous system diseases and can be adapted to profile exosomes from other somatic cell types, including astrocytes and microglia.

Expanding the capabilities of plasmonic biosensing to the single-particle level, Yang et al. introduced an interferometric plasmonic microscopy (iPM) system that enables label-free imaging and quantitative analysis of individual exosomes [64]. The platform provided high-resolution size profiling, revealing a continuous distribution with a peak around 62 nm—noticeably smaller than measurements obtained by NTA—underscoring the importance of calibration for precise sizing. Beyond morphological characterization, the iPM system was employed to investigate membrane fusion events between exosomes and liposomes, a key mechanism relevant to drug delivery. Fusion was confirmed by an observed increase in particle diameter following co-incubation. Critically, the system enabled real-time monitoring of binding events between exosomes and antibody-functionalized surfaces. A distinct “hit–stay–run” binding pattern was observed on antibody-coated substrates, contrasting with the Brownian motion seen on PEG-coated controls. Quantitative analysis of these dynamics provided insights into the binding energy landscape between exosomes and their cognate antibodies. Collectively, these findings highlight the iPM platform as a powerful tool for high-resolution, label-free analysis of exosome size, biophysical behavior, and surface interactions—supporting both fundamental studies in extracellular vesicle biology and future applications in clinical diagnostics and targeted delivery systems.

To further extend the analytical versatility of SPR-based platforms, Wu et al. developed an enhanced SPRi biosensor incorporating a Au-on-Ag heterostructured substrate for the ultrasensitive detection of exosomal microRNAs (miRNAs) associated with non-small cell lung cancer (NSCLC) [65]. The sensor achieved a remarkable detection limit of 1.68 fM, with a broad dynamic range spanning from 2 fM to 20 nM. The use of the Au-on-Ag nanostructure significantly amplified the SPR signal compared to conventional gold or silver surfaces, illustrating the advantage of plasmonic material engineering in boosting biosensor performance. Importantly, the platform demonstrated excellent antifouling properties, enabling consistent operation in complex biological fluids such as serum. This feature is essential for the accurate analysis of exosomes in minimally processed clinical samples. The biosensor was applied to plasma-derived exosomes from NSCLC patients and healthy individuals, successfully distinguishing between the two groups based on miRNA expression profiles. The system also supports multiplexed detection, allowing multiple miRNA targets to be profiled simultaneously from a single sample. This study highlights the potential of integrating nanostructured plasmonic substrates with SPRi for high-sensitivity, high-specificity exosomal miRNA profiling. Such platforms are particularly well suited for non-invasive, early-stage cancer diagnostics and exemplify the growing role of SPRi biosensors in liquid biopsy applications.

Demonstrating the multifunctionality of SPR-based biosensors, Yang et al. developed a SPR microscopy (SPRM) system capable of comprehensive extracellular vesicle (EV) analysis, including both exosomes and microvesicles [66]. The platform enabled accurate size profiling—reporting an average EV diameter of ~234 nm, consistent with NTA—and exhibited a robust linear correlation between EV concentration and SPR signal, supporting its suitability for high-throughput quantification. In addition to physical characterization, the SPRM system allowed for real-time monitoring of molecular interactions between EVs and capture antibodies. By analyzing binding and unbinding kinetics, the authors determined the equilibrium dissociation constant for CD63-EV interactions, offering quantitative insight into binding affinity. This dual capability underscores SPRM’s potential not only for vesicle enumeration and sizing but also for investigating biophysical interaction parameters. Overall, this work highlights SPRM as a versatile and accessible platform for the in-depth characterization of EVs, supporting both diagnostic applications and basic research on vesicle-mediated communication and disease mechanisms.

In the field of prostate cancer diagnostics, Chen et al. developed a SPRi biosensor that employed hydrogel–gold nanoparticle (AuNP) supramolecular spheres to enhance the capture and detection of exosomes derived from LNCaP prostate cancer cells [67]. The sensor demonstrated a wide linear detection range from 1 × 10^5^ to 1 × 10^7^ particles/mL, with a detection limit of 1 × 10^5^ particles/mL. Notably, SPRi signals showed strong correlation with prostate-specific antigen (PSA) levels obtained via chemiluminescence immunoassay, suggesting high potential for clinical translation. The biosensor also exhibited excellent specificity, as signal enhancement was observed exclusively in response to prostate cancer-derived exosomes, effectively discriminating them from other cell-line-derived vesicles. As a complementary approach, Wang et al. introduced a dual AuNP-assisted SPR aptasensor that utilized aptamer-modified nanoparticles to selectively bind and quantify cancer-associated exosomes [53]. This sensor achieved a remarkably low detection limit of 5 × 10^3^ exosomes/mL, substantially outperforming traditional ELISA-based techniques. The system successfully distinguished exosomes from MCF-7 breast cancer cells and MCF-10A normal cells, based on surface marker expression, and demonstrated strong reproducibility and regeneration capacity. Its performance was further validated using clinical serum samples, where the SPR response correlated well with PSA values, reinforcing its suitability for exosome-based liquid biopsy. Together, these studies exemplify the adaptability of SPR and SPRi platforms through the use of different recognition elements—antibodies and aptamers—offering sensitive, specific, and clinically relevant strategies for the detection and monitoring of prostate cancer-associated exosomes.

Highlighting the expanding role of aptamer-based interfaces in plasmonic biosensing, Liao et al. developed a surface plasmon resonance (SPR) assay for the sensitive and specific detection of exosomes derived from hepatic carcinoma SMMC-7721 cells [68]. The platform utilized AuNPs functionalized with polydopamine (Au@PDA NPs), synthesized through dopamine polymerization on AuNP surfaces, to amplify signal output and improve target capture efficiency. Aptamers targeting hepatic tumor exosomes were immobilized on the sensor surface, and their binding interactions with exosomes induced significant SPR signal shifts, confirming successful and specific recognition. The assay achieved a detection limit of 5.6 × 10^5^ exosomes/mL, demonstrating enhanced sensitivity relative to many existing SPR-based platforms. Specificity was confirmed through comparative analysis against exosomes from non-cancerous cell lines (e.g., HepG2 and MCF-7), where only the SMMC-7721-derived vesicles produced pronounced responses. Notably, the platform operated effectively in 50% fetal bovine serum without requiring extensive sample pretreatment, underscoring its robustness in biologically relevant matrices. This study underscores the versatility of aptamer-functionalized nanostructures in SPR biosensing and highlights their applicability in exosome-based liquid biopsy. The demonstrated ability to distinguish cancer-specific exosomes in complex biological media reinforces their suitability for non-invasive diagnostics and longitudinal disease monitoring.

Chen et al. developed a label-free SPR biosensor tailored for the sensitive and specific detection of HER2-positive exosomes, a clinically relevant biomarker for breast cancer diagnosis and treatment monitoring [58]. The sensing mechanism is based on a non-enzymatic signal amplification strategy utilizing tyramine-coated AuNPs (AuNPs-Ty) in the presence of hydrogen peroxide (H_2_O_2_), which enables selective deposition of nanoparticles on the surface of HER2-positive exosomes. This interaction significantly enhances the SPR signal, allowing for improved sensitivity. The biosensor demonstrated a linear detection range from 1.0 × 10^4^ to 1.0 × 10^7^ particles/mL and exhibited strong specificity, as HER2-negative exosomes produced substantially lower signal intensities. Clinical validation using serum samples from breast cancer patients and healthy individuals revealed consistently higher SPR signals in the patient group, confirming the platform’s diagnostic relevance. This study not only highlights the clinical utility of HER2-positive exosome profiling via SPR but also introduces an innovative amplification approach that bypasses enzymatic steps, simplifying assay workflow. The incorporation of molecular aptamer beacon (MAB) technology further enhances specificity, offering a promising route for rapid and robust liquid biopsy platforms in breast cancer and potentially other exosome-related malignancies.

**Figure 2:**
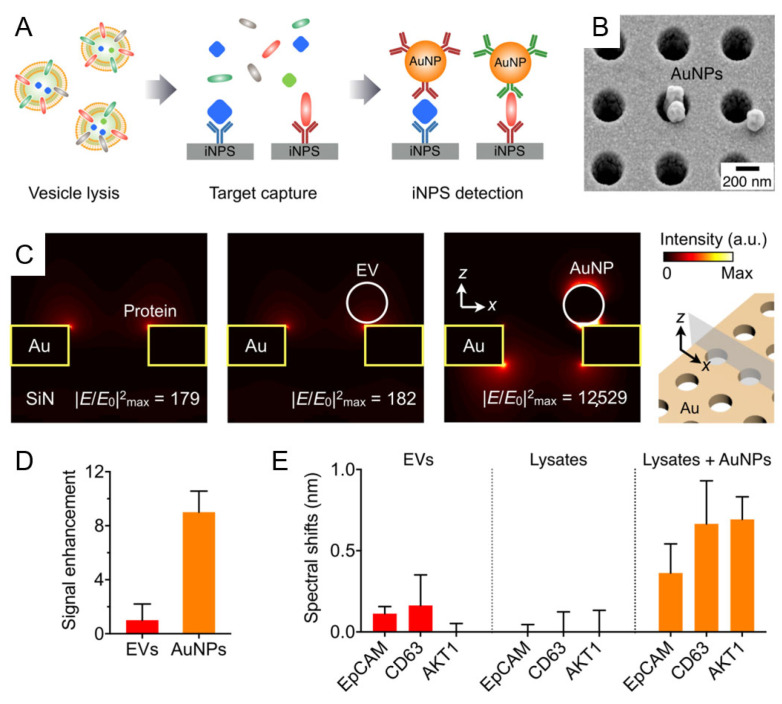
Intravesicular nanoplasmonic system (iNPS) for enhanced detection of exosomal proteins. (**A**) Schematic representation of the iNPS assay workflow. Exosomes are lysed to release intravesicular and transmembrane proteins, which are captured on the plasmonic chip and labeled with AuNPs for signal amplification. (**B**) Scanning electron micrograph showing AuNPs on the iNPS nanohole array. (**C**) FDTD simulations comparing electromagnetic field intensities for protein, EV, and AuNP binding. AuNP labeling achieved a ~70-fold field enhancement relative to native binding. (**D**) Signal enhancement comparison between EVs and AuNPs, demonstrating ~9-fold greater spectral shift with AuNP-labeled targets. (**E**) Validation of multiplexed detection of transmembrane (EpCAM, CD63) and intravesicular (AKT1) proteins using the iNPS platform. Reprinted with permission from Ref. [69]. Copyright (2018) American Chemical Society.

To address the challenge of profiling both surface and intravesicular exosomal proteins, Park et al. developed the intravesicular nanoplasmonic system (iNPS) based on nanohole array-enabled SPR sensing [69] (Figure 2). Unlike conventional SPR biosensors limited to surface markers, the iNPS platform integrates vesicle lysis, immunocapture, and nanoparticle-enhanced signal amplification to enable detection of internal cargo proteins and achieve comprehensive molecular profiling of cancer-derived exosomes. The system utilizes a 10 × 10 nanohole array format for high-throughput, multiplexed detection with minimal sample volume (0.5 µL per marker). As shown in Figure 2A, lysed exosomes expose both transmembrane and intravesicular proteins, which are selectively captured and labeled with 100 nm AuNPs to amplify the SPR response. Figure 2B presents an SEM image of AuNP-labeled nanohole arrays, while Figure 2C shows FDTD simulation results indicating a ~70-fold enhancement in localized electromagnetic fields. This results in a ~9-fold increase in spectral shift compared to native vesicle binding (Figure 2D). The platform successfully detected transmembrane proteins (e.g., CD63, EpCAM) as well as intravesicular proteins such as AKT1 (Figure 2E), with expression profiles that closely mirrored those of the originating tumor cells. This correlation underscores the clinical relevance of the platform and its potential applications in cancer subtype classification, treatment monitoring, and liquid biopsy-based biomarker discovery. Overall, iNPS represents a notable advancement in SPR-based exosome biosensing by extending detection beyond surface-level targets, though further clinical validation is warranted to confirm its diagnostic and prognostic utility in real-world settings.

Recent advances in nanomaterial engineering have significantly enhanced the performance of SPR-based biosensors for exosome detection. Qiu et al. introduced a titanium nitride (TiN)-based SPR platform functionalized with biotinylated antibodies (BAF-TiN) for the sensitive detection of glioma-derived exosomal proteins, achieving detection limits of 4.29 × 10^−3^ µg/mL for CD63 and 2.75 × 10^−3^ µg/mL for the epidermal growth factor receptor variant-III (EGFRvIII), a glioma-specific mutant protein [70]. The platform demonstrated a broad dynamic range and maintained analytical reliability in complex matrices, including mouse serum samples, supporting its potential for non-invasive glioma diagnostics. As an alternative material strategy, Wang et al. employed a two-dimensional metal–organic framework (2D MOF) based on copper-tetra(4-carboxyphenyl) porphyrin (Cu-TCPP) to develop a plasmonic biosensor for the detection of PD-L1-positive exosomes [71]. The Cu-TCPP 2D MOF offered tunable optical properties, biocompatibility, and signal amplification capacity. The biosensor achieved an exceptionally low detection limit of 16.7 particles/mL and showed consistent recovery rates (93.4–102.4%) in spiked human serum samples, underscoring its analytical robustness and clinical utility. Complementing this approach, Mao et al. reported a graphene-modified SPR sensor integrated with multifunctional antifouling peptides (M-Pep) for the ultrasensitive detection of PD-L1-expressing exosomes [72]. This system achieved a detection limit of 20 particles/mL and maintained signal integrity in complex biological environments. The antifouling performance of the M-Pep coating was validated in serum, minimizing nonspecific binding and enabling reliable signal readout. Collectively, these studies demonstrate how material innovation—including TiN, 2D MOFs, and graphene-based hybrid systems—can be strategically leveraged to improve the sensitivity, specificity, and clinical applicability of SPR biosensors. Each platform offers a unique route for enhancing signal transduction and overcoming biological matrix interference, positioning these next-generation materials as key enablers in the development of exosome-based liquid biopsy technologies.

### 5.2. LSPR-Based Platforms

LSPR-based biosensors have emerged as powerful tools for exosome detection, offering excellent sensitivity, scalability, and compatibility with compact diagnostic platforms. These systems exploit localized electromagnetic fields generated at the surface of metallic nanostructures, allowing for precise detection of refractive index changes induced by biomolecular interactions. Recent advances in nanomaterials, microfluidics, and data analysis have further enhanced their performance across diverse clinical applications.

**Figure 3:**
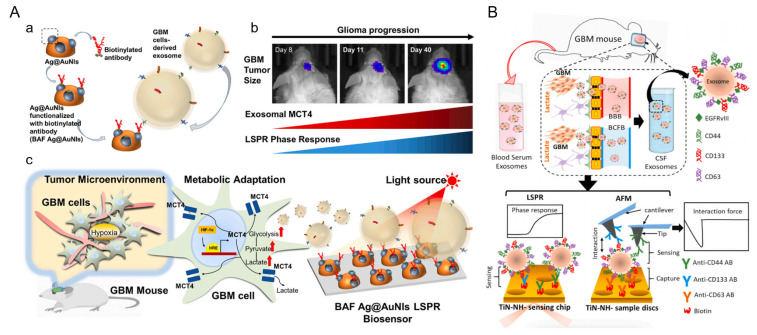
Schematic overview of label-free nanoplasmonic and AFM-based biosensing platforms for exosomal biomarker detection in glioblastoma (GBM). (**A**) BAF Ag@AuNIs-based LSPR biosensor for the detection of MCT4-enriched exosomes secreted by hypoxic GBM cells. (**a**) Schematic illustration of the sensor chip with site-specific biotinylated anti-MCT4 antibody functionalization. (**b**,**c**) Hypoxia-induced glioma progression leads to increased MCT4 expression and exosome release, which correlate with enhanced LSPR phase response. Reprinted with permission from Ref. [56]. Copyright (2022) Elsevier B.V. (**B**) Dual biosensing strategy employing TiN–nanohole-based LSPR and TIC-AFM enables multiplexed detection of CD44 and CD133 in blood and CSF exosomes from a GBM mouse model. Lactate-induced malignancy elevates these exosomal markers, which are sensitively captured and quantified for non-invasive monitoring. Reprinted with permission from Ref. [73]. Copyright (2021) Elsevier B.V.

In the context of glioblastoma diagnostics, two distinct LSPR platforms have demonstrated significant potential. Liu et al. introduced a biosensor integrating silver nanoparticles on gold nano-islands (Ag@AuNIs) functionalized with biotinylated antibodies to detect MCT4, a hypoxia-related marker associated with glioma progression [56]. As illustrated in Figure 3A, metabolic reprogramming of GBM cells under hypoxic conditions leads to increased MCT4 expression and exosome secretion. These exosomal MCT4 molecules are sensitively detected using a biotinylated antibody-functionalized Ag@Au nanostructure-based biosensor (BAF Ag@AuNIs LSPR). The LSPR platform is designed with site-specific antibody immobilization to ensure high specificity and signal amplification, achieving a low detection limit of 1.4 ng/mL. Importantly, the system effectively differentiated glioma-derived exosomes in both cultured cells and mouse serum samples, confirming that exosomal MCT4 levels closely correlate with tumor malignancy. Similarly, Thakur et al. reported a titanium nitride (TiN)-nanohole LSPR sensor for the detection of glioma markers CD44 and CD133. Their study demonstrated that lactate-stimulated glioma cells exhibited enhanced CD44 expression and exosome secretion, which in turn promoted tumor cell migration and angiogenesis. The TiN–NH-LSPR platform achieved a detection limit of 3.46 × 10^−3^ μg/mL for exosomal CD44, outperforming the TIC-AFM-based method (LOD: 5.29 × 10^−1^ μg/mL). Notably, the LSPR sensor successfully detected elevated levels of CD44 and CD133 in blood and CSF from a glioblastoma mouse model, highlighting its clinical potential for minimally invasive, multi-biomarker liquid biopsy diagnostics in GBM monitoring. As illustrated in Figure 3B, this dual biosensing strategy enables multiplexed, label-free detection using LSPR phase shifts and AFM-based interaction forces, offering a powerful tool for early diagnosis and molecular monitoring of glioblastoma (GBM) [73].

Li et al. developed an optical microfiber-based LSPR biosensor enhanced with molybdenum diselenide (MoSe_2_)-supported gold nanorods for the ultrasensitive detection of CA9-positive exosomes associated with clear cell renal carcinoma [74]. The sensor exhibited a strong linear correlation between exosome concentration and red shift in transmission spectra, achieving a remarkably low detection limit of 9.32 particles/mL. Validated using patient-derived plasma samples, the biosensor effectively distinguished cancer-derived exosomes from normal ones and correlated CA9 expression levels with disease progression. These findings underscore its potential for non-invasive clinical diagnostics using exosome-based liquid biopsy.

Lv et al. developed a low-cost, label-free plasmonic biosensor array integrated with microfluidics for the detection of CD63-positive exosomes [75]. The biosensor utilized gold nano-ellipsoid arrays fabricated via anodic aluminum oxide templates and functionalized with anti-CD63 antibodies. By monitoring shifts in extinction spectra as exosome samples flowed through the microfluidic system, the platform achieved a detection limit of 1 ng/mL. This setup enabled rapid, efficient, and scalable exosome detection, with potential for multiplexed biomarker analysis through simple surface modifications.

In the field of neurodegenerative diagnostics, two innovative platforms demonstrated strong promise for AD detection through exosome analysis. Lim et al. introduced the Amplified Plasmonic Exosome (APEX) platform to differentiate exosome-bound from unbound amyloid-beta (Aβ) species in blood samples [59]. The sensor achieved a sensitivity of approximately 200 exosomes and showed that Aβ42+CD63+ exosomes correlated more strongly with PET imaging than free Aβ, underscoring their potential as reflective biomarkers of AD pathology. Complementarily, Song et al. developed a DNA-assembled plasmonic biosensor (DAPA) to detect exosomal miRNAs (exo-miR-125b and exo-miR-361) in AD patient serum [76]. The DAPA platform achieved attomolar-level detection with 91.67% sensitivity and 99.52% diagnostic accuracy, highlighting its applicability for early-stage AD diagnosis.

In cancer diagnostics, structurally advanced plasmonic platforms have demonstrated high sensitivity and adaptability. Zhu et al. reported a 3D plasmonic photonic crystal (PPC) biosensor that combined LSPR with Fabry–Perot resonances, achieving a sensitivity of 1376 nm/RIU and a wide dynamic detection range of 1 × 10^4^ to 1 × 10^11^ particles/mL [77]. Spectral shifts from 9 to 102 nm confirmed its quantitative precision for cancer-related exosome analysis. Similarly, Amrhein et al. introduced Dual Imaging Single-Vesicle Technology (DISVT) for profiling surface markers on individual EVs, with a particular focus on HER2-positive breast cancer [78]. DISVT integrated dual-mode fluorescence and light scattering imaging with automated analysis, enabling early detection of HER2+ EVs and distinguishing early from advanced-stage patients, demonstrating its utility for real-time cancer monitoring.

Together, these studies illustrate the broad clinical potential of LSPR-based and vesicle-resolved biosensors, particularly when combined with advanced nanostructures, optical modalities, and automated analysis. These integrated approaches offer powerful solutions for the sensitive and specific detection of exosomal biomarkers in both oncology and neurodegenerative disease diagnostics.

### 5.3. SERS-Based Platforms

Building upon the SERS principles outlined earlier, this section highlights representative applications of SERS-based biosensors in exosome diagnostics, focusing on cancer detection, drug delivery profiling, and machine learning integration.

**Figure 4:**
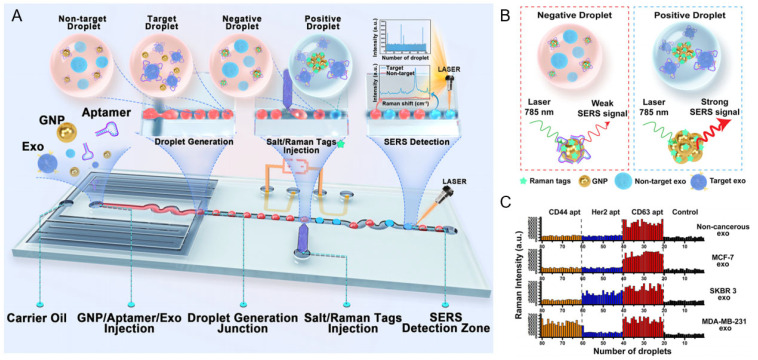
Schematic overview and multiplexed detection performance of a SERS-based droplet microfluidic platform for HER2-positive exosome identification. (**A**) Illustration of the overall workflow. Individual droplets containing HER2 aptamer-functionalized AuNPs and exosomes are generated and injected with salt and Raman tags. Upon target exosome recognition, aptamer detachment triggers AuNP aggregation, leading to strong SERS signal generation at the detection zone. (**B**) Mechanism of SERS signal generation. Positive droplets containing HER2-positive exosomes induce salt-mediated AuNP aggregation and hot spot formation, resulting in strong SERS signals, while negative droplets yield minimal signal. (**C**) Multiplexed detection results for various exosome types (non-cancerous, MCF-7, SKBR3, MDA-MB-231) using aptamers against CD44, HER2, and CD63. High Raman intensities are observed specifically for SKBR3 exosomes with HER2 aptamers, demonstrating aptamer specificity and clinical relevance. Reprinted with permission from Ref. [57]. Copyright (2024) American Chemical Society.

A representative clinical application of nanoplasmonic biosensing is the SERS-based droplet microfluidic platform developed by Ho et al. for high-throughput detection of HER2-positive exosomes [57] (Figure 4A). In this system, salt-induced aggregation of HER2 aptamer-functionalized AuNPs generated strong SERS signals within target-containing droplets, enabling single-droplet level detection with an LOD of 4.5 particles/μL. The DTNB SERS signal at 1339 cm^−1^ increased proportionally with SKBR3 exosome concentration, whereas HER2-negative and non-cancerous exosomes yielded negligible signals. Notably, multiplexed aptamer screening enabled reliable identification even at 0.001% target abundance, reflecting the system’s high sensitivity and selectivity. Clinical validation using plasma samples further confirmed that HER2-positive patients exhibited significantly stronger SERS responses than HER2-negative or healthy controls (Figure 4B). In comparative profiling across various exosome types using different aptamers, the CD63 aptamer produced uniformly high Raman signals, consistent with its role as a general exosome marker. In contrast, the HER2 aptamer selectively generated strong signals only in response to SKBR3-derived exosomes, demonstrating high target specificity for HER2-positive cancer detection (Figure 4C). Principal component analysis (PCA) of the SERS spectra revealed clear clustering according to HER2 status, underscoring the platform’s diagnostic accuracy and translational potential for liquid biopsy-based cancer diagnostics.

**Figure 5:**
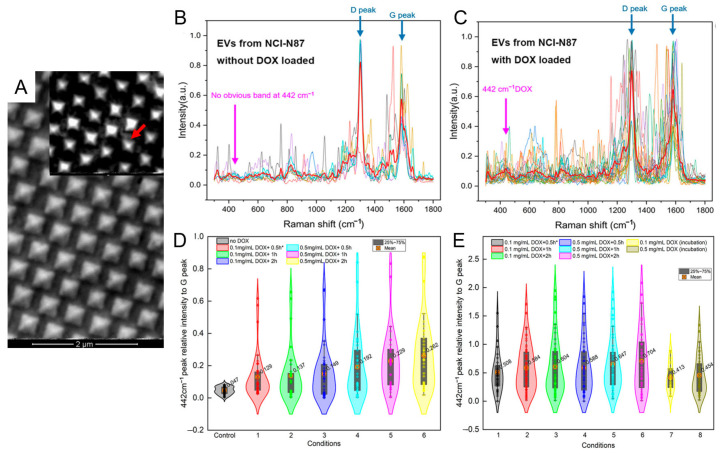
SERS-based single-exosome quantification of doxorubicin (DOX) loading using graphene-coated gold nanopyramid substrates. (**A**) SEM image of the gold nanopyramid array SERS substrate. The inset highlights transferred single-layer graphene coverage with visible surface wrinkles (red arrow), which serves as an internal calibration layer for Raman intensity. (**B**,**C**) Superimposed SERS spectra of individual exosomes derived from the NCI-N87 cell line without (**B**) and with (**C**) DOX loading. The 442 cm^−1^ DOX peak is selectively present only in drug-loaded samples, with clear D and G peaks from graphene. (**D**) Violin plot showing relative 442 cm^−1^/G peak intensities in DOX-loaded exosomes from the NCI-N87 line under different incubation conditions. (**E**) Violin plot showing relative 442 cm^−1^/G peak intensities in DOX-loaded exosomes from A549 cells treated under hypotonic conditions with varying DOX concentrations and durations. * EVs were derived from NCI-N87 cell line. The times refer to the durations of the incubation of DOX with EVs. Reprinted with permission from Ref. [79]. Copyright (2025) MDPI.

Expanding the application scope, Liu et al. employed a graphene-coated gold nanopyramid array for SERS-based quantification of doxorubicin (DOX) loading within individual exosomes [79]. As shown in Figure 5A, the nanopyramid surface was uniformly covered with monolayer graphene to serve as an internal Raman reference. Superimposed SERS spectra of DOX-loaded versus unloaded exosomes derived from the NCI-N87 cell line (Figure 5B,C) showed a pronounced increase in the 442 cm^−1^ Raman peak, reflecting successful drug incorporation. Violin plots of the intensity ratio between the 442 cm^−1^ and graphene G peak (Figure 5D,E) revealed heterogeneous drug-loading efficiencies across exosome populations. Application of hypotonic pretreatment significantly enhanced loading—raising incorporation efficiency from 23.0% to 55.1% depending on exposure time. Comparative analysis further indicated that A549-derived exosomes responded more strongly to osmotic stimulation than those from NCI-N87, highlighting cell-type-dependent loading dynamics. These findings underscore the critical role of osmotic modulation in cargo loading and validate the system’s ability to quantify therapeutic payloads at the single-vesicle level. Such high-resolution quantification is pivotal for optimizing exosome-based drug delivery in personalized cancer nanomedicine.

**Figure 6:**
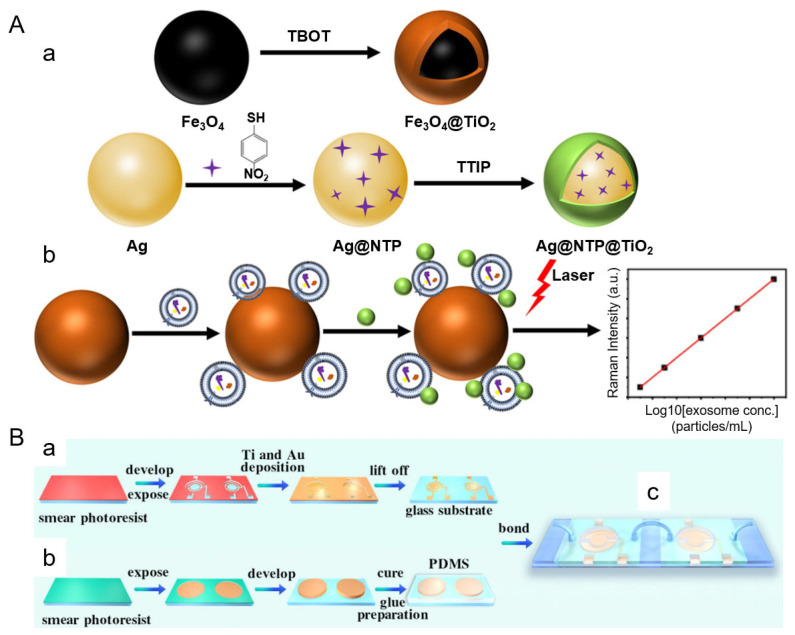
(**A**) Nanomaterial synthesis and exosome detection workflow (**a**) Fe_3_O_4_@TiO_2_ magnetic nanoparticles are synthesized for exosome capture under an external magnetic field, while Ag@NTP@TiO_2_ core-shell SERS tags enable stable and sensitive Raman signal enhancement for quantitative detection. (**b**) Exosomes are labeled with SERS tags and analyzed by laser excitation, producing concentration-dependent Raman signals. (**B**) Microfluidic chip fabrication process. (**a**) Patterned microelectrodes are prepared using a lift-off photolithographic process. (**b**) PDMS-based microfluidic channels are molded, aligned, and bonded to the electrode substrate. (**c**) The final integrated chip incorporates magnetic bead-based exosome capture, DNA cascade amplification, and SERS detection zones within a dual-chamber architecture. (**A**,**B**) are reprinted with permission from Ref. [80] and Ref. [81], respectively. Copyright (2025) American Chemical Society.

Several recent studies have highlighted the potential of integrating SERS with exosome enrichment and signal amplification strategies to facilitate clinical translation. Zheng et al. developed a rapid, cost-effective platform that combines titanium dioxide (TiO_2_)-coated magnetic nanoparticles (Fe_3_O_4_@TiO_2_) for exosome isolation with Ag@NTP@TiO_2_ SERS tags for sensitive, label-free detection [77]. As shown in Figure 6A, the magnetic nanoparticles enabled ultrafast exosome capture under an external magnetic field, followed by rapid labeling with TiO_2_-shelled SERS tags that stabilized the silver core and enhanced Raman signals. This antibody-free approach significantly reduced assay complexity and achieved a detection limit of 640 particles/mL within 10 min. The system demonstrated consistent signal intensity across a wide dynamic range (1.9 × 10^3^ to 3.8 × 10^7^ particles/mL) and achieved an area under the curve (AUC) of 0.88 in plasma-based ROC analysis, indicating strong diagnostic performance. Additionally, it exhibited high capture efficiency (up to 90%) and excellent reproducibility with minimal interference from biological matrices.

Complementing this magnetic nanoparticle-based strategy, Ma et al. developed a dual-chamber microfluidic platform that integrates on-chip magnetic bead-based exosome isolation with downstream DNA cascade amplification and SERS detection [78]. As illustrated in Figure 6B, the chip consists of a magnetic separation chamber where aptamer-functionalized beads capture target exosomes and an amplification chamber that uses an alternating current electric field to enhance DNA-triggered signal generation. The resulting SERS signals provide highly sensitive readouts, achieving a detection limit of 10.9 particles/μL and excellent linearity (*R*^2^ > 0.997) across a range of 100 to 2.4 × 10^6^ particles/μL. Specificity was validated using cell-line-derived exosomes, and recovery rates in spiked serum samples ranged from 95.94% to 102.67%.

Advancements in SERS-based biosensing have opened new avenues for the ultrasensitive detection and molecular profiling of cancer-derived exosomes. By integrating nanostructured materials, surface functionalization strategies, and signal amplification techniques, these platforms have demonstrated remarkable performance in clinical diagnostics and liquid biopsy applications.

One such approach employed polydopamine-modified substrates to construct a multilayered SERS nano-tag, enabling the detection of a single pancreatic cancer-derived exosome from just 2 mL of sample solution [82]. This immunoassay demonstrated a sensitivity of 95.7% for identifying metastatic tumors and differentiating tumor-node-metastasis (TNM) stages. By analyzing clinical serum samples, the platform not only enabled early-stage diagnosis but also supported real-time monitoring of tumor progression, offering a powerful tool for non-invasive cancer classification.

Wang et al. reported a complementary strategy using magnetic gold-shell nanobeads conjugated with CD63-targeting aptamers to capture exosomes, while AuNPs carrying Raman reporters served as SERS probes [83]. The binding of exosomes led to a measurable decrease in SERS intensity in the supernatant, indicating successful capture. The detection limits reached 32, 73, and 203 exosomes/μL for SKBR3, T84, and LNCaP exosomes, respectively. Validation with blood samples from cancer patients demonstrated the method’s potential for early screening and clinical utility in multiplexed cancer detection.

Pang et al. advanced this concept further by developing a personalized detection platform for circulating exosomal PD-L1 using Fe_3_O_4_@TiO_2_ nanoparticles [84]. The TiO_2_ surface selectively captured exosomes via interactions with the hydrophilic phosphate heads of phospholipid membranes, achieving a 96.5% capture efficiency within 5 min. Exosomal PD-L1 was labeled with Au@Ag@MBA SERS tags, allowing quantification down to 1 PD-L1^+^ exosome/μL. This enabled precise differentiation between NSCLC patients and healthy individuals, providing insights into disease staging and potential responses to immunotherapy.

Focusing on prostate cancer, Kim et al. developed a label-free SERS sensor capable of detecting urinary exosomal miRNAs using a three-dimensional hierarchical plasmonic nanoarchitecture [85]. This configuration created dense plasmonic hotspots, allowing the detection of miR-10a and miR-21 at levels as low as 10 aM with high selectivity. Clinical validation demonstrated a diagnostic accuracy of 0.93, underscoring the platform’s potential in early cancer diagnosis and real-time biomarker monitoring for personalized medicine.

Meanwhile, Lin et al. engineered a porous-plasmonic SERS chip functionalized with CP05 polypeptide to directly isolate CD63^+^ exosomes from blood samples [86]. Enhanced SERS signals enabled detection of tissue inhibitor of metalloproteinases-1 (TIMP-1), a protein associated with cancer progression. The platform achieved a sensitivity of 32 exosomes/μL and, when coupled with machine learning, reached an identification accuracy of 85.72%, demonstrating its promise for early diagnosis of lung and colon cancers.

Lastly, Li et al. introduced a hierarchical SERS (H-SERS) platform based on magnetic bead-exosome-SERS probe assemblies (MEDP) for the sensitive detection of pancreatic cancer biomarkers LRG1 and GPC1 [87]. This system reached a detection limit of 15 particles/μL and achieved an AUC of 0.95 in early-stage diagnosis. By integrating proteomic profiling with SERS readouts, the platform offered a comprehensive solution for high-performance cancer screening.

Recent advances in exosome biosensing have increasingly focused on combining nanoplasmonic techniques with AI to extract meaningful biological insights from complex datasets. Among these, label-free SERS platforms offer unique advantages for single-exosome analysis, as they capture subtle biochemical variations without the need for external labeling or prior biomarker knowledge.

## 6. Deep Learning-Enhanced SERS Platforms for Label-Free Exosome Profiling

**Figure 7:**
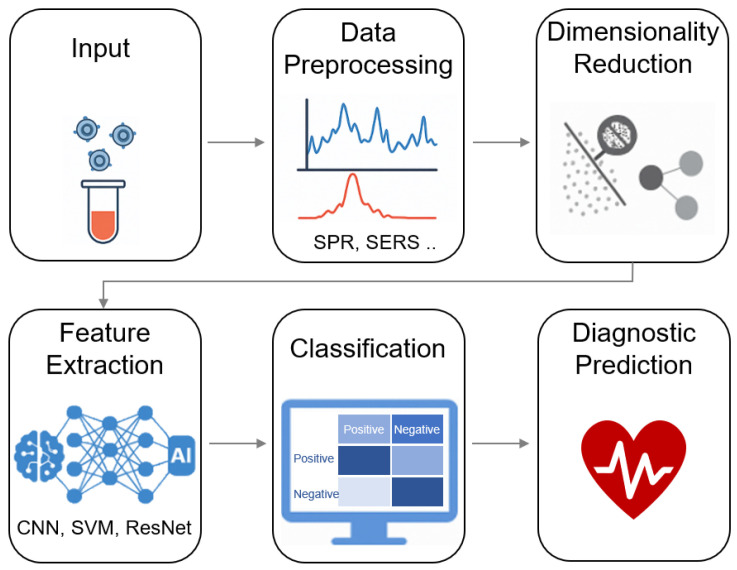
AI-assisted biosensing workflow for exosome analysis. The schematic illustrates key steps in integrating artificial intelligence with nanoplasmonic exosome biosensing, including signal acquisition, data preprocessing, dimensionality reduction, feature extraction, and final classification. This pipeline enables label-free, high-throughput, and accurate disease profiling based on exosomal signatures.

The integration of AI with nanoplasmonic biosensors—particularly SERS—has advanced label-free, non-invasive molecular profiling of exosomes. As illustrated in Figure 7, AI-enhanced biosensing workflows typically include several key stages: signal acquisition (e.g., Raman spectra), data preprocessing (e.g., baseline correction and normalization), dimensionality reduction (e.g., PCA or t-SNE), and feature extraction and classification using machine learning or deep learning models such as SVMs, CNNs, and ResNets. These systems enable high-throughput identification of exosomal subtypes and disease signatures without prior biomarker selection.

However, it is important to critically assess the generalizability of these AI models. Most published studies have trained and tested their models on limited datasets, typically involving exosomes derived from only three to five cell lines or small numbers of clinical samples, as observed in the examples listed in Table 2. While these studies often report high classification accuracy (frequently above 90%), the use of small and homogeneous datasets increases the risk of overfitting and restricts the clinical applicability of the models. External validation on larger, more diverse cohorts remains rare, limiting confidence in the ability of these models to generalize to real-world clinical samples.

**Figure 8:**
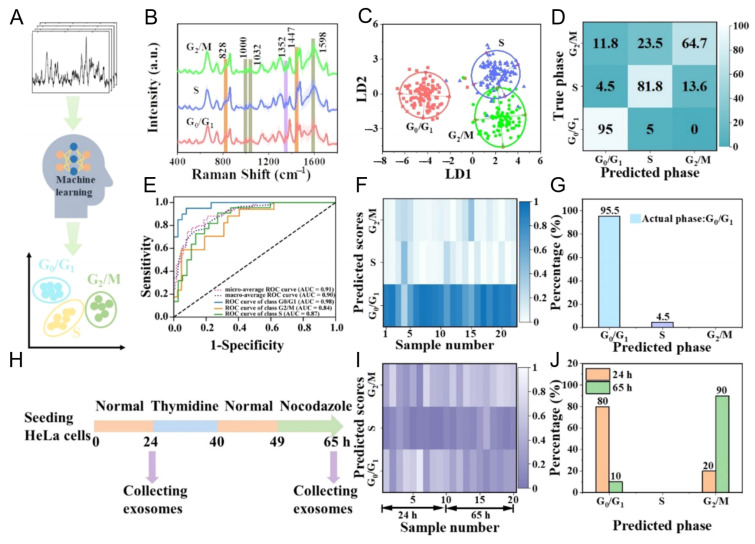
AI-assisted classification of cell cycle stages based on label-free SERS spectra of exosomes derived from HeLa cells. (**A**) Schematic illustration of the machine learning framework used to distinguish exosomes originating from G_0_/G_1_, S, and G_2_/M phase-arrested cells. (**B**) Average SERS spectra showing distinct vibrational features among the three cell cycle stages. (**C**,**D**) Linear discriminant analysis (LDA) score plot and support vector machine (SVM) confusion matrix validating classification accuracy. (**E**) Receiver operating characteristic (ROC) curves demonstrating the predictive performance of the SVM model. (**F**,**G**) Predicted distribution of cell cycle phases in exosomes from asynchronously cultured HeLa cells at 24 h, analyzed using the trained model. (**H**) Conceptual scheme for time-resolved SERS analysis of exosomes collected at 24 and 65 h. (**I**,**J**) Model-predicted cell cycle phase distributions at each time point, reflecting a dynamic shift from G_0_/G_1_ to G_2_/M. Reprinted with permission from Ref. [88]. Copyright (2025) American Chemical Society.

A representative example is the study by Diao et al., who employed a PCA–SVM framework to classify exosomes corresponding to different cell cycle stages, achieving high discrimination accuracy. Specifically, their study developed a machine learning-assisted SERS strategy to classify exosomes derived from HeLa cells arrested at distinct cell cycle stages (G_0_/G_1_, S, and G_2_/M) [88] (Figure 8). By recording intrinsic Raman spectral signatures from individual exosomes, the system enabled data-driven classification through linear discriminant analysis (LDA) and support vector machine (SVM) models. These models effectively distinguished phase-specific exosome populations, revealing distinct biochemical fingerprints associated with each cell cycle stage. Importantly, the method was validated using time-course cultures of asynchronously growing cells. The resulting exosome profiles accurately reflected dynamic shifts in the cell cycle—indicating a predominance of G_0_/G_1_ phase at 24 h and a transition toward G_2_/M phase at 65 h. This suggests that exosomes can encode and transmit physiological state information from their parent cells, and that machine learning algorithms can decode this information from label-free SERS data. The integration of AI with nanoplasmonic platforms not only enhances diagnostic resolution but also opens up new possibilities for functional profiling of exosomes in regenerative medicine, oncology, and drug response monitoring. As demonstrated in this study, such systems can serve as non-invasive “exosomal fingerprints” of cellular function, enabling real-time molecular phenotyping without prior biomarker selection.

Chen et al. utilized a CNN-based approach integrated with a microfluidic-SERS platform to classify NSCLC subtypes, reporting an accuracy of 95–97%. Their study developed a non-invasive, accurate, and rapid method for early diagnosis and molecular subtyping of NSCLC by characterizing exosome heterogeneity using an integrated microfluidic platform combining label-free SERS and deep learning [89]. The authors designed a microfluidic chip capable of capturing and enriching exosomes from cell culture media using polystyrene microspheres conjugated with gold nanocubes and anti-CD-9 antibodies (PACD), followed by SERS signal acquisition to provide molecular fingerprints of the exosomes. The SERS spectra were then analyzed using a custom deep learning model based on a residual neural network (ResNet) architecture, enabling classification of exosomes derived from three NSCLC cell lines (NCI-H460, NCI-H226, and PC-9) and a normal lung epithelial cell line (BEAS-2B). Key results demonstrated that the platform achieved a high exosome trapping efficiency of up to 85%, and the deep learning model distinguished NSCLC subtypes from normal controls with an overall accuracy of 97.88% and an AUC exceeding 0.95 for each category. These findings highlight the potential of integrating microfluidics, SERS, and deep learning for exosome-based, non-invasive cancer diagnostics and precise molecular subtyping, paving the way for future clinical applications in personalized medicine for lung cancer patients.

In a similar context, Lu et al. developed a convolutional neural network (CNN)-SVM hybrid model for early-stage lung cancer diagnosis using plasma-derived exosomes [90]. The CNN was trained on SERS spectral data from exosomes derived from lung cancer cell lines (NCI-H226, HCC-827, A549) and normal lung epithelial cells (BEAS-2B). Subsequently, the authors employed a SVM model to extract and classify clinical plasma exosomes as features. The platform’s capacity to differentiate between adenocarcinoma in situ (AIS) and healthy controls was demonstrated by key results, which included an AUC of 0.84, 83.3% sensitivity, and 83.3% specificity. This outperformed conventional methods such as PCA-LDA (AUC 0.63) and PLS-DA (AUC 0.8). The CNN was able to classify cell-line-derived exosomes with 100% accuracy, and the integrated AI-SERS approach effectively addressed the heterogeneity challenges associated with exosomes in clinical samples. This work provides a prospective alternative to invasive tissue biopsies for early cancer diagnosis by integrating exosome molecular profiling with AI-driven pattern recognition, thereby establishing a framework for liquid biopsy applications.

Ma et al. developed a label-free, non-invasive platform for early breast cancer detection by integrating SERS with a ResNet-based CNN to analyze exosomes derived from normal (MCF-10A) and malignant (MCF-7, MDA-MB-231) breast cell lines [91]. Exosomes were isolated via ultracentrifugation, and their Raman signals were enhanced using AuNPs, enabling acquisition of molecular fingerprints across the 600–1800 cm^−1^ spectral range. A deep learning framework was trained on 1160 SERS spectra preprocessed with baseline correction, denoising, and min–max normalization to classify exosome subtypes. The CNN achieved 95% classification accuracy with AUC values of 0.999 (MDA-MB-231), 0.991 (MCF-7), and 0.997 (MCF-10A), significantly outperforming principal component analysis (PCA), which showed overlapping confidence ellipses and reduced sensitivity (56.3–89.7%). Distinct SERS peaks at 939 cm^−1^ (proline C=C stretching), 1145 cm^−1^ (protein CH vibrations), and 1380 cm^−1^ (nucleotides) were identified, with cancer exosomes exhibiting reduced lipid/protein signal intensities compared to normal counterparts. This work establishes a rapid, cost-effective framework for exosome-based liquid biopsies, underscoring the clinical potential of AI-enhanced SERS analysis in breast cancer screening.

Jalali et al. introduced a highly sensitive, label-free approach for molecular profiling of single EVs using a novel multiplex fluidic device integrated with MoS_2_-plasmonic nanocavity microchips (MoSERS microchip), specifically for the early detection and monitoring of GBM [92]. The method involves fabricating arrayed plasmonic nanocavities with an embedded MoS_2_ monolayer. This unique design enables robust, label-free isolation and nanoconfinement of single EVs (achieving 97% confinement with minimal fluid volumes), leveraging physical interactions between the MoS_2_ edge sites and the EV lipid bilayer. Simultaneously, the layered plasmonic cavity ensures significant electromagnetic field enhancement, enabling single-EV level SERS signal resolution for detailed molecular fingerprinting. Applying this system to the GBM paradigm, the researchers successfully identified and distinguished signals from single EVs derived from non-cancerous glial cells, various glioma cell lines, and glioma stem cells, including those exhibiting critical molecular alterations such as EGFRvIII oncogenic mutations and MGMT expression. A key result highlighted a detection limit of 1.23% for stratifying these molecular variants within a wild-type EV population. Furthermore, when interfaced with the CNN, the MoSERS system demonstrated an improved diagnostic accuracy of 87% for detecting GBM mutations in 12 patient blood samples, a performance comparable to traditional clinical pathology tests. This innovative work underscores the significant potential of plasmonic nanostructures combined with advanced machine learning for highly sensitive, real-time molecular stratification of cancer patients using circulating EVs.

Another notable study by Shin et al. focused on the early-stage diagnosis of lung cancer through a deep learning-based spectroscopic analysis of circulating exosomes, which is highly relevant to plasmonic nanostructures for exosome biosensing [93]. Their aim was to provide an accurate diagnosis of early-stage lung cancer by analyzing the SERS signals of human plasma-derived exosomes. The method involved isolating exosomes from both cell culture supernatants (from normal and lung cancer cell lines) and human plasma samples (from healthy controls and lung cancer patients). These exosomes were characterized using NTA and Western blotting to confirm their exosomal nature. SERS signals were then collected from these exosomes deposited on AuNP-coated plates. A ResNet-based deep learning model was trained using the SERS data from cell-derived exosomes to classify cell types with high accuracy. This pre-trained model was then used to analyze human plasma exosomes by calculating their “relative similarity” to cancer cell exosomes, leveraging features extracted from the hidden layers of the neural network and Mahalanobis distance for quantification. Key results demonstrated that the deep learning model, trained with cell-derived SERS signals, could classify these exosomes with 95% accuracy. Crucially, in a cohort of 43 lung cancer patients (including Stage I and II), the model predicted that plasma exosomes from 90.7% of patients showed a higher similarity to lung cancer cell exosomes compared to healthy controls, with this similarity correlating with cancer progression. The method achieved an impressive AUC of 0.912 for the entire cohort and 0.910 for Stage I patients, demonstrating its potential for early-stage liquid biopsy in lung cancer.

Together, these studies illustrate the tremendous diagnostic potential of SERS-based biosensors in capturing and analyzing exosomes across a wide range of cancer types. The integration of engineered nanomaterials, rapid enrichment strategies, and advanced signal amplification techniques not only enhances detection sensitivity and specificity, but also supports the development of real-time, non-invasive diagnostics tailored to individual patient needs. Collectively, the convergence of nanoplasmonics and AI is poised to transform exosome-based diagnostics, enabling highly personalized and data-driven clinical applications.

## 7. Future Directions and Challenges

While nanoplasmonic biosensors have demonstrated remarkable sensitivity and specificity for exosome detection, several unresolved challenges continue to hinder their clinical translation. A fundamental issue is the biological heterogeneity of exosomes: they are secreted by diverse cell types, and even individual cells can produce multiple subpopulations. This complexity complicates the accurate identification of the cellular origin of exosomes, which is critical for their diagnostic interpretation.

In addition, analytical outcomes are highly sensitive to pre-analytical variables such as sample heterogeneity, collection methods, storage conditions, and processing time. These factors highlight the urgent need for standardized repositories, rigorous calibration protocols, and cross-laboratory benchmarking to ensure reproducibility and comparability across studies [94].

From a materials standpoint, the variability and high surface reactivity of nanomaterials often compromise signal stability and detection reproducibility. Standardized synthesis protocols and quality control measures are required to address these limitations. Moreover, the intrinsic toxicity of many nanomaterials remains a significant concern for in vivo applications. Research into biocompatible alternatives and toxicity mitigation strategies must be prioritized [95]. Importantly, systematic studies on long-term stability, biodistribution, metabolic fate, and residual accumulation of nanomaterials within biological systems are lacking.

Another important consideration is the potential alteration of exosomal cargo by the local microenvironment created by nanostructured materials or detection surfaces. These interactions may affect molecular composition or bias analytical outcomes. To overcome such issues, strategies for non-disruptive capture and controlled release, such as stimuli-responsive cleavage mechanisms, should be further developed [96]. Furthermore, the lack of standardized quantification units across studies complicates the comparison of detection limits and performance metrics. Establishing universal units for exosome concentration and signal reporting would improve clarity and consistency in the field [97].

Emerging trends highlight the integration of multiple sensing modalities and the development of intelligent, programmable nanostructures. Coupling computational design with advanced material engineering could enable tunable, adaptive platforms offering enhanced performance and predictability. Concurrently, increased investment is needed to improve the portability, repeatability, and user-friendliness of bioanalytical devices, especially for clinical and POC applications. In particular, simulation-guided nanomaterial development may accelerate the creation of highly predictable, application-specific platforms [98].

In summary, addressing these biological, material, and analytical challenges is essential to bridge the gap between proof-of-concept demonstrations and real-world clinical implementation of nanoplasmonic biosensors for exosome analysis and therapy.

## 8. Conclusions

Nanoplasmonic biosensors have emerged as powerful tools for exosome analysis, offering high sensitivity, label-free operation, and multiplexed capabilities. This review has highlighted recent advances in plasmonic substrate engineering, optical detection schemes, and clinical applications of PSPR, LSPR, and SERS-based platforms.

While considerable progress has been made, successful clinical implementation will depend on addressing key limitations, including assay standardization, substrate reproducibility, and the need for robust, automated data analysis. Integration with microfluidics and AI represents a promising direction to streamline workflow and enhance performance.

Looking ahead, nanoplasmonic platforms are well-positioned to revolutionize exosome-based diagnostics. Their ability to provide real-time, non-invasive molecular insights aligns with the growing demand for personalized medicine. Continued interdisciplinary collaboration between materials science, engineering, and clinical research will be crucial to transition these technologies from the laboratory to the clinic.

## Figures and Tables

**Table 1:** Comparative summary of PSPR, LSPR, and SERS for plasmonic biosensing applications.

Parameter	PSPR	LSPR	SERS
Electromagnetic Field Penetration	200–300 nm	10–30 nm	1–3 nm (localized hotspots)
Surface Sensitivity	Moderate	High	Extremely high
Signal Enhancement	10^0^–10^2^	10^2^–10^4^	10^6^–10^8^
Reproducibility	High (planar metal films)	Moderate (depends on nanostructure uniformity)	Low (depends on hotspot uniformity)
Integration with Portable Devices	Challenging	Easily miniaturized	Moderate
Fabrication Complexity	Low (thin-film deposition)	Moderate to high (nanoarrays)	High (nanogap/nanostructure patterning)
Detection Format	Angle/length shift	Spectral shift/colorimetric change	Raman spectral fingerprint
Typical Use Case	Bulk protein interaction, serum analysis	Surface protein detection on nanoscale targets	Single-molecule or exosome detection
Limit of Detection	1–10 ng/mL	10–100 pg/mL	1–10 fg/mL
Sensitivity	Moderate	High	Very High
Specificity	Moderate to High	High	High
Sample Type	Serum, plasma	Exosome isolates, urine	Plasma, single EVs

**Table 2:** Representative studies of AI-integrated SERS platforms for exosome-based and cell-derived disease diagnostics.

SERS Platform	Al/ML Algorithm	Target Disease	Dataset Size (Spectra/Samples)	Cell Lines/Clinical Samples	Reported Accuracy/AUC	Ref.
Label-free SERS	PCA + SVM	Cell cycle stage discrimination	~300 spectra	3 (HeLa G_0_/G_1_, S, G_2_/M)	LDA/SVM: >90%	[88]
Microfluidic SERS	ResNet	NSCLC subtype classification	4 cell lines; 85% trapping efficiency	3 NSCLC (NCI-H460, H226, and PC-9), 1 normal (BEAS-2B)	ResNet: 97.88%, AUC > 0.95	[89]
Plasma-based SERS	1D-CNN	Early-stage lung cancer detection	Not specified (cell lines + plasma)	3 lung cancers (NCI-H226, HCC-827, and A549), 1 normal (BEAS-2B); plasma samples	CNN: 100% (cell lines), AUC 0.84 (clinical)	[90]
Label-free SERS	ResNet	Breast cancer subtype classification	1160 spectra	2 breast cancers (MCF-7 and MDA-MB-231), 1 normal (MCF-10A)	CNN: 95%, AUC > 0.99	[91]
MoS_2_ nanocavity-enhanced single-EV SERS	ResNet	Glioblastoma progression profiling	12 patient blood samples; single EVs	Non-cancerous glial, glioma, and glioma stem cells; GBM patients	CNN: 87% (clinical)	[92]
Spectroscopic SERS	ResNet	Early-stage lung cancer diagnosis	43 plasma samples	3 lung cancers, 1 normal (cell lines + plasma)	AUC: 0.912 (clinical)	[93]

## Data Availability

Not applicable.

## References

[1] Pegtel D.M., Gould S.J. (2019). Exosomes. Annu. Rev. Biochem..

[2] Thery C., Ostrowski M., Segura E. (2009). Membrane Vesicles as Conveyors of Immune Responses. Nat. Rev. Immunol..

[3] Urbanelli L., Magini A., Buratta S., Brozzi A., Sagini K., Polchi A., Tancini B., Emiliani C. (2013). Signaling Pathways in Exosomes Biogenesis, Secretion and Fate. Genes.

[4] Wu J., Huang J., Yu J., Xu M., Liu J., Pu K. (2024). Exosome-Inhibiting Polymeric Sonosensitizer for Tumor-Specific Sonodynamic Immunotherapy. Adv. Mater..

[5] Essola J.M., Zhang M., Yang H., Li F., Xia B., Mavoungou J.F., Hussain A., Huang Y. (2024). Exosome Regulation of Immune Response Mechanism: Pros and Cons in Immunotherapy. Bioact. Mater..

[6] Sun T., Li M., Liu Q., Yu A., Cheng K., Ma J., Murphy S., McNutt P.M., Zhang Y. (2024). Insights into Optimizing Exosome Therapies for Acute Skin Wound Healing and Other Tissue Repair. Front. Med..

[7] Zhang Y., Fang M., Zhu J., Li T., Li N., Su B., Sun G.D., Li L., Zhou C. (2024). Exosome-loaded Hyaluronic Acid Hydrogel Composite with Oxygen-producing 3D Printed Polylactic Acid Scaffolds for Bone Tissue Repair and Regeneration. Int. J. Biol. Macromol..

[8] Shao M., Gao Y., Xu X., Chan D.W., Du J. (2024). Exosomes: Key Factors in Ovarian Cancer Peritoneal Metastasis and Drug Resistance. Biomolecules.

[9] Huda M.N., Nafiujjaman M., Deaguero I.G., Okonkwo J., Hill M.L., Kim T., Nurunnabi M. (2021). Potential Use of Exosomes as Diagnostic Biomarkers and in Targeted Drug Delivery: Progress in Clinical and Preclinical Applications. ACS Biomater. Sci. Eng..

[10] Wang Z., Wang Q., Qin F., Chen J. (2024). Exosomes: A Promising Avenue for Cancer Diagnosis beyond Treatment. Front. Cell Dev. Biol..

[11] Nishimura H., Hashii N., Yamamoto T., Sun Y., Miura T., Sato Y., Ishii-Watabe A. (2024). Usefulness of Size-Exclusion Chromatography-Multi-Angle Light Scattering to Assess Particle Composition and Protein Impurities for Quality Control of Therapeutic Exosome Preparations. Pharmaceutics.

[12] Kowkabany G., Bao Y. (2024). Nanoparticle Tracking Analysis: An Effective Tool to Characterize Extracellular Vesicles. Molecules.

[13] Tian Y., Yan X., Wang Q., Zheng L. (2024). Flow Cytometry for Single Extracellular Vesicle Analysis. Extracellular Vesicles.

[14] Liu Z., Xue H., Chen Q., Yang G. (2023). A Method for Extraction of Exosomes from Breast Tumour Cells and Characterisation by Transmission Electron Microscopy. J. Microsc..

[15] Chelnokova I.A., Nikitina I.A., Starodubtseva M.N. (2024). Mechanical Properties of Blood Exosomes and Lipoproteins after the Rat Whole Blood Irradiation with X-rays in Vitro Explored by Atomic Force Microscopy. Micron.

[16] Skliar M., Chernyshev V.S. (2019). Imaging of Extracellular Vesicles by Atomic Force Microscopy. J. Vis. Exp..

[17] Bairamukov V.Y., Bukatin A.S., Kamyshinsky R.A., Burdakov V.S., Pichkur E.B., Shtam T.A., Starodubtseva M.N. (2022). Nanomechanical Characterization of Exosomes and Concomitant Nanoparticles from Blood Plasma by PeakForce AFM in Liquid. Biochim. Biophys. Acta Gen. Subj..

[18] Sbarigia C., Tacconi S., Mura F., Rossi M., Dinarelli S., Dini L. (2022). High-resolution Atomic Force Microscopy as a Tool for Topographical Mapping of Surface Budding. Front. Cell Dev. Biol..

[19] Sajidah E.S., Lim K., Yamano T., Nishide G., Qiu Y., Yoshida T., Wang H., Kobayashi A., Hazawa M., Dewi F.R.P. (2022). Spatiotemporal Tracking of Small Extracellular Vesicle Nanotopology in Response to Physicochemical Stresses Revealed by HS-AFM. J. Extracell. Vesicles.

[20] Mathew B., Mansuri M.S., Williams K.R., Nairn A.C. (2021). Exosomes as Emerging Biomarker Tools in Neurodegenerative and Neuropsychiatric Disorders-A Proteomics Perspective. Brain Sci..

[21] Zhang Y., Bi J., Huang J., Tang Y., Du S., Li P. (2020). Exosome: A Review of Its Classification, Isolation Techniques, Storage, Diagnostic and Targeted Therapy Applications. Int. J. Nanomed..

[22] Hessvik N.P., Llorente A. (2018). Current Knowledge on Exosome Biogenesis and Release. Cell. Mol. Life Sci..

[23] Sergazy S., Adekenov S., Khabarov I., Adekenova K., Maikenova A., Aljofan M. (2025). Harnessing Mammalian- and Plant-Derived Exosomes for Drug Delivery: A Comparative Review. Int. J. Mol. Sci..

[24] He C., Hua W., Liu J., Fan L., Wang H., Sun G. (2020). Exosomes Derived from Endoplasmic Reticulum-stressed Liver Cancer Cells Enhance the Expression of Cytokines in Macrophages via the STAT3 Signaling Pathway. Oncol. Lett..

[25] Li D., Wang Y., Jin X., Hu D., Xia C., Xu H., Hu J. (2020). NK Cell-derived Exosomes Carry miR-207 and Alleviate Depression-like Symptoms in Mice. J. Neuroinflamm..

[26] Zhao D., Yu Z., Li Y., Wang Y., Li Q., Han D. (2020). GelMA Combined with Sustained Release of HUVECs Derived Exosomes for Promoting Cutaneous Wound Healing and Facilitating Skin Regeneration. J. Mol. Histol..

[27] Kalluri R., LeBleu V.S. (2020). The Biology, Function, and Biomedical Applications of Exosomes. Science.

[28] Kanchanapally R., Deshmukh S.K., Chavva S.R., Tyagi N., Srivastava S.K., Patel G.K., Singh A.P., Singh S. (2019). Drug-loaded Exosomal Preparations from Different Cell Types Exhibit Distinctive Loading Capability, Yield, and Antitumor Efficacies: A Comparative Analysis. Int. J. Nanomed..

[29] Agrawal A.K., Aqil F., Jeyabalan J., Spencer W.A., Beck J., Gachuki B.W., Alhakeem S.S., Oben K., Munagala R., Bondada S. (2017). Milk-derived Exosomes for Oral Delivery of Paclitaxel. Nanomedicine.

[30] Schwarz G., Ren X., Xie W., Guo H., Jiang Y., Zhang J. (2025). Engineered Exosomes: A Promising Drug Delivery Platform with Therapeutic Potential. Front. Mol. Biosci..

[31] Fan X., Zhang Y., Liu W., Shao M., Gong Y., Wang T., Xue S., Nian R. (2024). A comprehensive Review of Engineered Exosomes from the Preparation Strategy to Therapeutic Applications. Biomater. Sci..

[32] Si C., Gao J., Ma X. (2024). Engineered Exosomes in Emerging Cell-free Therapy. Front. Oncol..

[33] Zhang M., Hu S., Liu L., Dang P., Liu Y., Sun Z., Qiao B., Wang C. (2023). Engineered Exosomes from Different Sources for Cancer-targeted Therapy. Signal Transduct. Target. Ther..

[34] Ahmadi S.E., Soleymani M., Shahriyary F., Amirzargar M.R., Ofoghi M., Fattahi M.D., Safa M. (2023). Viral Vectors and Extracellular Vesicles: Innate Delivery Systems Utilized in CRISPR/Cas-mediated Cancer Therapy. Cancer Gene Ther..

[35] Dai Z., Cai R., Zeng H., Zhu H., Dou Y., Sun S. (2024). Exosome May Be the Next Generation of Promising Cell-free Vaccines. Hum. Vaccin. Immunother..

[36] Balaraman A.K., Babu M.A., Moglad E., Mandaliya V., Rekha M.M., Gupta S., Prasad G.V.S., Kumari M., Chauhan A.S., Ali H. (2025). Exosome-mediated Delivery of CRISPR-Cas9: A Revolutionary Approach to Cancer Gene Editing. Pathol. Res. Pract..

[37] Antimisiaris S.G., Mourtas S., Marazioti A. (2018). Exosomes and Exosome-Inspired Vesicles for Targeted Drug Delivery. Pharmaceutics.

[38] Kowal E.J.K., Ter-Ovanesyan D., Regev A., Church G.M. (2017). Extracellular Vesicle Isolation and Analysis by Western Blotting. Methods Mol. Biol..

[39] Lee J., Kim H., Heo Y., Yoo Y.K., Han S.I., Kim C., Hur D., Kim H., Kang J.Y., Lee J.H. (2019). Enhanced Paper-based ELISA for Simultaneous EVs/exosome Isolation and Detection Using Streptavidin Agarose-based Immobilization. Analyst.

[40] Brown B.A., Zeng X., Todd A.R., Barnes L.F., Winstone J.M.A., Trinidad J.C., Novotny M.V., Jarrold M.F., Clemmer D.E. (2020). Charge Detection Mass Spectrometry Measurements of Exosomes and other Extracellular Particles Enriched from Bovine Milk. Anal. Chem..

[41] Dragovic R.A., Gardiner C., Brooks A.S., Tannetta D.S., Ferguson D.J., Hole P., Carr B., Redman C.W., Harris A.L., Dobson P.J. (2011). Sizing and Phenotyping of Cellular Vesicles Using Nanoparticle Tracking Analysis. Nanomedicine.

[42] Bachurski D., Schuldner M., Nguyen P.H., Malz A., Reiners K.S., Grenzi P.C., Babatz F., Schauss A.C., Hansen H.P., Hallek M. (2019). Extracellular Vesicle Measurements with Nanoparticle Tracking Analysis—An Accuracy and Repeatability Comparison between NanoSight NS300 and ZetaView. J. Extracell. Vesicles.

[43] Nurrohman D.T., Chiu N.F., Hsiao Y.S., Lai Y.J., Nanda H.S. (2024). Advances in Nanoplasmonic Biosensors: Optimizing Performance for Exosome Detection Applications. Biosensors.

[44] Butt M.A. (2025). Surface Plasmon Resonance-Based Biodetection Systems: Principles, Progress and Applications—A Comprehensive Review. Biosensors.

[45] Hu Y., Wang Y., Zhang Y., Yang H. (2024). Recent Advances in Plasmonic Sensing Techniques for Exosome Detection and Composition Analysis. Laser Photon. Rev..

[46] Mcoyi M.P., Mpofu K.T., Sekhwama M., Mthunzi-Kufa P. (2024). Developments in Localized Surface Plasmon Resonance. Plasmonics.

[47] Ryu J.-H., Lee H.Y., Lee J.-Y., Kim H.-S., Kim S.-H., Ahn H.S., Ha D.H., Yi S.N. (2021). Enhancing SERS Intensity by Coupling PSPR and LSPR in a Crater Structure with Ag Nanowires. Appl. Sci..

[48] Yizhao P., Fang C., Yuchang L., Wenxing Y., Zao Y., Shaolin K. (2024). Coherent Coupling of Localized Surface Plasmons and Surface Plasmons in Borophene-based Metamaterial. Micro Nanostructures.

[49] Sina A.A., Vaidyanathan R., Wuethrich A., Carrascosa L.G., Trau M. (2019). Label-free Detection of Exosomes Using a Surface Plasmon Resonance Biosensor. Anal. Bioanal. Chem..

[50] Picciolini S., Gualerzi A., Vanna R., Sguassero A., Gramatica F., Bedoni M., Masserini M., Morasso C. (2018). Detection and Characterization of Different Brain-Derived Subpopulations of Plasma Exosomes by Surface Plasmon Resonance Imaging. Anal. Chem..

[51] Nanda B.P., Rani P., Paul P., Aman, Ganti S.S., Bhatia R. (2024). Recent Trends and Impact of Localized Surface Plasmon Resonance (LSPR) and Surface-enhanced Raman Spectroscopy (SERS) in Modern Analysis. J. Pharm. Anal..

[52] Wang W., You Y., Gunasekaran S. (2021). LSPR-based Colorimetric Biosensing for Food Quality and Safety. Compr. Rev. Food. Sci. Food Saf..

[53] Wang Q., Zou L., Yang X., Liu X., Nie W., Zheng Y., Cheng Q., Wang K. (2019). Direct Quantification of Cancerous Exosomes via Surface Plasmon Resonance with Dual Gold Nanoparticle-assisted Signal Amplification. Biosens. Bioelectron..

[54] Zhang H., Zhou X., Li X., Gong P., Zhang Y., Zhao Y. (2023). Recent Advancements of LSPR Fiber-Optic Biosensing: Combination Methods, Structure, and Prospects. Biosensors.

[55] Min J., Son T., Hong J.S., Cheah P.S., Wegemann A., Murlidharan K., Weissleder R., Lee H., Im H. (2020). Plasmon-Enhanced Biosensing for Multiplexed Profiling of Extracellular Vesicles. Adv. Biosyst..

[56] Liu L.L., Thakur A., Li W.K., Qiu G.Y., Yang T., He B., Lee Y.J., Wu C.M.L. (2022). Site Specific Biotinylated Antibody Functionalized Ag@AuNIs LSPR Biosensor for the Ultrasensitive Detection of Exosomal MCT4, a Glioblastoma Progression Biomarker. Chem. Eng. J..

[57] Ho K.H.W., Lai H., Zhang R., Chen H., Yin W., Yan X., Xiao S., Lam C.Y.K., Gu Y., Yan J. (2024). SERS-Based Droplet Microfluidic Platform for Sensitive and High-Throughput Detection of Cancer Exosomes. ACS Sens..

[58] Chen W., Li Z., Cheng W., Wu T., Li J., Li X., Liu L., Bai H., Ding S., Li X. (2021). Surface Plasmon Resonance Biosensor for Exosome Detection Based on Reformative Tyramine Signal Amplification Activated by Molecular Aptamer Beacon. J. Nanobiotechnol..

[59] Lim C.Z.J., Zhang Y., Chen Y., Zhao H., Stephenson M.C., Ho N.R.Y., Chen Y., Chung J., Reilhac A., Loh T.P. (2019). Subtyping of Circulating Exosome-bound Amyloid Beta Reflects Brain Plaque Deposition. Nat. Commun..

[60] Xie Y., Su X., Wen Y., Zheng C., Li M. (2022). Artificial Intelligent Label-Free SERS Profiling of Serum Exosomes for Breast Cancer Diagnosis and Postoperative Assessment. Nano Lett..

[61] Song S., Lee J.U., Jeon M.J., Kim S., Lee C.-N., Sim S.J. (2023). Precise Profiling of Exosomal Biomarkers via Programmable Curved Plasmonic Nanoarchitecture-Based Biosensor for Clinical Diagnosis of Alzheimer’s Disease. Biosens. Bioelectron..

[62] Wu X., Zhao H., Natalia A., Lim C.Z.J., Ho N.R.Y., Ong C.-A.J., Teo M.C.C., So J.B.Y., Shao H. (2020). Exosome-Templated Nanoplasmonics for Multiparametric Molecular Profiling. Sci. Adv..

[63] Im H., Shao H., Park Y.I., Peterson V.M., Castro C.M., Weissleder R., Lee H. (2014). Label-free Detection and Molecular Profiling of Exosomes with a Nano-plasmonic Sensor. Nat. Biotechnol..

[64] Yang Y., Shen G., Wang H., Li H., Zhang T., Tao N., Ding X., Yu H. (2018). Interferometric Plasmonic Imaging and Detection of Single Exosomes. Proc. Natl. Acad. Sci. USA.

[65] Wu W., Yu X., Wu J., Wu T., Fan Y., Chen W., Zhao M., Wu H., Li X., Ding S. (2021). Surface Plasmon Resonance Imaging-based Biosensor for Multiplex and Ultrasensitive Detection of NSCLC-associated Exosomal miRNAs Using DNA Programmed Heterostructure of Au-on-Ag. Biosens. Bioelectron..

[66] Yang Y., Zhai C., Zeng Q., Khan A.L., Yu H. (2020). Multifunctional Detection of Extracellular Vesicles with Surface Plasmon Resonance Microscopy. Anal. Chem..

[67] Chen W., Li J., Wei X., Fan Y., Qian H., Li S., Xiang Y., Ding S. (2020). Surface Plasmon Resonance Biosensor Using Hydrogel-AuNP Supramolecular Spheres for Determination of Prostate Cancer-derived Exosomes. Microchim. Acta.

[68] Liao G., Liu X., Yang X., Wang Q., Geng X., Zou L., Liu Y., Li S., Zheng Y., Wang K. (2020). Surface Plasmon Resonance Assay for Exosomes Based on Aptamer Recognition and Polydopamine-functionalized Gold Nanoparticles for Signal Amplification. Microchim. Acta.

[69] Park J., Im H., Hong S., Castro C.M., Weissleder R., Lee H. (2018). Analyses of Intravesicular Exosomal Proteins Using a Nano-Plasmonic System. ACS Photonics.

[70] Qiu G., Thakur A., Xu C., Ng S.P., Lee Y., Wu C.M.L. (2018). Detection of Glioma-Derived Exosomes with the Biotinylated Antibody-Functionalized Titanium Nitride Plasmonic Biosensor. Adv. Funct. Mater..

[71] Wang Y., Mao Z., Chen Q., Koh K., Hu X., Chen H. (2022). Rapid and Sensitive Detection of PD-L1 Exosomes Using Cu-TCPP 2D MOF as a SPR Sensitizer. Biosens. Bioelectron..

[72] Mao Z.H., Zhao J.L., Chen J., Hu X.J., Koh K., Chen H.X. (2021). A Simple and Direct SPR Platform Combining Three-in-one Multifunctional Peptides for Ultra-sensitive Detection of PD-L1 Exosomes. Sens. Actuator B-Chem..

[73] Thakur A., Xu C., Li W.K., Qiu G., He B., Ng S.P., Wu C.L., Lee Y. (2021). In Vivo Liquid Biopsy for Glioblastoma Malignancy by the AFM and LSPR Based Sensing of Exosomal CD44 and CD133 in a Mouse Model. Biosens. Bioelectron..

[74] Li H., Huang T., Lu L., Yuan H., Zhang L., Wang H., Yu B. (2022). Ultrasensitive Detection of Exosomes Using an Optical Microfiber Decorated with Plasmonic MoSe(2)-Supported Gold Nanorod Nanointerfaces. ACS Sens..

[75] Lv X., Geng Z., Su Y., Fan Z., Wang S., Fang W., Chen H. (2019). Label-Free Exosome Detection Based on a Low-Cost Plasmonic Biosensor Array Integrated with Microfluidics. Langmuir.

[76] Song S., Lee J.U., Jeon M.J., Kim S., Sim S.J. (2022). Detection of Multiplex Exosomal miRNAs for Clinically Accurate Diagnosis of Alzheimer’s Disease Using Label-free Plasmonic Biosensor Based on DNA-assembled Advanced Plasmonic Architecture. Biosens. Bioelectron..

[77] Zhu S., Li H., Yang M., Pang S.W. (2018). Highly Sensitive Detection of Exosomes by 3D Plasmonic Photonic Crystal Biosensor. Nanoscale.

[78] Amrhein K., Taylor M.L., Wilson R., Gallops C.E., Annamer A., Vinduska V., Kwizera E.A., Zhang H., Wang Y., Hoang T.B. (2023). Dual Imaging Single Vesicle Surface Protein Profiling and Early Cancer Detection. ACS Appl. Mater. Interfaces.

[79] Liu J., Srivastava S., Li T., Moujane F., Lee J.Y., Chen Y., Liu H., Deng S.X., Xie Y.H. (2025). On the Feasibility of SERS-Based Monitoring of Drug Loading Efficiency in Exosomes for Targeted Delivery. Biosensors.

[80] Zheng S., Su N., Zhang R., Chen X., Zhang J., Gao M., Zhang X. (2025). A Surface-Enhanced Raman Scattering Platform for Rapid, Sensitive, and Cost-Effective Quantitative Analysis of Exosomes Based on Titanium Dioxide Functionalized Nanomaterials. Anal. Chem..

[81] Ma J., Li K., Duan Z., Yang X., Zhou G., Ye S. (2025). On-Chip Isolation and Reciprocal Signal Amplification Detection of Tumor-Derived Exosomes in Dual-Control Microfluidic Device. Anal. Chem..

[82] Li T.D., Zhang R., Chen H., Huang Z.P., Ye X., Wang H., Deng A.M., Kong J.L. (2018). An Ultrasensitive Polydopamine Bi-functionalized SERS Immunoassay for Exosome-based Diagnosis and Classification of Pancreatic Cancer. Chem. Sci..

[83] Wang Z., Zong S., Wang Y., Li N., Li L., Lu J., Wang Z., Chen B., Cui Y. (2018). Screening and Multiple Detection of Cancer Exosomes Using an SERS-based Method. Nanoscale.

[84] Pang Y., Shi J., Yang X., Wang C., Sun Z., Xiao R. (2020). Personalized Detection of Circling Exosomal PD-L1 Based on Fe_3_O_4_@TiO_2_ Isolation and SERS Immunoassay. Biosens. Bioelectron..

[85] Kim W.H., Lee J.U., Jeon M.J., Park K.H., Sim S.J. (2022). Three-dimensional Hierarchical Plasmonic Nano-architecture Based Label-free Surface-enhanced Raman Spectroscopy Detection of Urinary Exosomal miRNA for Clinical Diagnosis of Prostate Cancer. Biosens. Bioelectron..

[86] Lin C., Liang S., Li Y., Peng Y., Huang Z., Li Z., Yang Y., Luo X. (2022). Localized Plasmonic Sensor for Direct Identifying Lung and Colon Cancer from the Blood. Biosens. Bioelectron..

[87] Li J., Li Y., Chen S., Duan W., Kong X., Wang Y., Zhou L., Li P., Zhang C., Du L. (2022). Highly Sensitive Exosome Detection for Early Diagnosis of Pancreatic Cancer Using Immunoassay Based on Hierarchical Surface-Enhanced Raman Scattering Substrate. Small Methods.

[88] Diao X., Qi G., Li X., Tian Y., Li J., Jin Y. (2025). Label-Free Exosomal SERS Detection Assisted by Machine Learning for Accurately Discriminating Cell Cycle Stages and Revealing the Molecular Mechanisms during the Mitotic Process. Anal. Chem..

[89] Chen H., Liu H., Xing L., Fan D., Chen N., Ma P., Zhang X. (2025). Deep Learning-driven Microfluidic-SERS to Characterize the Heterogeneity in Exosomes for Classifying Non-Small Cell Lung Cancer Subtypes. ACS Sens..

[90] Lu D., Shangguan Z., Su Z., Lin C., Huang Z., Xie H. (2024). Artificial Intelligence-based Plasma Exosome Label-free SERS Profiling Strategy for Early Lung Cancer Detection. Anal. Bioanal. Chem..

[91] Ma X., Xiong H., Guo J., Liu Z., Han Y., Liu M., Guo Y., Wang M., Zhong H., Guo Z. (2022). Label-free Breast Cancer Detection and Classification by Convolutional Neural Network-based on Exosomes Surface-enhanced Raman scattering. J. Innov. Opt. Health Sci..

[92] Jalali M., Del Real Mata C., Montermini L., Jeanne O., Hosseini I.I., Gu Z., Spinelli C., Lu Y., Tawil N., Guiot M.C. (2023). MoS_2_-Plasmonic Nanocavities for Raman Spectra of Single Extracellular Vesicles Reveal Molecular Progression in Glioblastoma. ACS Nano.

[93] Shin H., Oh S., Hong S., Kang M., Kang D., Ji Y.G., Choi B.H., Kang K.W., Jeong H., Park Y. (2020). Early-Stage Lung Cancer Diagnosis by Deep Learning-Based Spectroscopic Analysis of Circulating Exosomes. ACS Nano.

[94] Witwer K.W., Buzás E.I., Bemis L.T., Bora A., Lässer C., Lötvall J., Nolte-‘t Hoen E.N., Piper M.G., Sivaraman S., Skog J. (2013). Standardization of Sample Collection, Isolation and Analysis Methods in Extracellular Vesicle Research. J. Extracell. Vesicles.

[95] Cheng N., Du D., Wang X., Liu D., Xu W., Luo Y., Lin Y. (2019). Recent Advances in Biosensors for Detecting Cancer-Derived Exosomes. Trends Biotechnol..

[96] Shao H., Im H., Castro C.M., Breakefield X., Weissleder R., Lee H. (2018). New Technologies for Analysis of Extracellular Vesicles. Chem. Rev..

[97] Jackman J.A., Rahim Ferhan A., Cho N.-J. (2017). Nanoplasmonic Sensors for Biointerfacial Science. Chem. Soc. Rev..

[98] Patel M.T., Goldberg Oppenheimer P.G. (2025). Advancements in Cancer Diagnostics: Integrating Surface-enhanced Raman Spectroscopy and Microptofluidics for Precision and Versatility. Appl. Spectrosc. Rev..

